# F‐actin dynamics in midgut cells enables virus persistence in vector insects

**DOI:** 10.1111/mpp.13260

**Published:** 2022-09-08

**Authors:** Hui Wang, Yan Liu, Wenwen Liu, Kongming Wu, Xifeng Wang

**Affiliations:** ^1^ State Key Laboratory for Biology of Plant Diseases and Insect Pests Institute of Plant Protection, Chinese Academy of Agricultural Sciences Beijing China; ^2^ State Key Laboratory of Ecological Pest Control for Fujian and Taiwan Crops Fujian Agriculture and Forestry University Fuzhou China

**Keywords:** F‐actin dynamics, geminivirus, hemipteran, luteovirus, persistent transmission

## Abstract

Hemipteran insects that transmit plant viruses in a persistent circulative manner acquire, retain and transmit viruses for their entire life. The mechanism enabling this persistence has remained unclear for many years. Here, we determined how wheat dwarf virus (WDV) persists in its leafhopper vector *Psammotettix alienus*. We found that WDV caused the up‐regulation of actin‐depolymerizing factor (ADF) at the mRNA and protein levels in the midgut cells of leafhoppers after experiencing a WDV acquisition access period (AAP) of 6, 12 or 24 h. Experimental inhibition of F‐actin depolymerization by jasplakinolide and dsRNA injection led to lower virus accumulation levels and transmission efficiencies, suggesting that depolymerization of F‐actin regulated by ADF is essential for WDV invasion of midgut cells. Exogenous viral capsid protein (CP) inhibited ADF depolymerization of actin filaments in vitro and in *Spodoptera frugiperda* 9 (Sf9) cells because the CP competed with actin to bind ADF and then blocked actin filament disassembly. Interestingly, virions colocalized with ADF after a 24‐h AAP, just as actin polymerization occurred, indicating that the binding of CP with ADF affects the ability of ADF to depolymerize F‐actin, inhibiting WDV entry. Similarly, the luteovirus barley yellow dwarf virus also induced F‐actin depolymerization and then polymerization in the gut cells of its vector *Schizaphis graminum*. Thus, F‐actin dynamics are altered by nonpropagative viruses in midgut cells to enable virus persistence in vector insects.

## Graphical abstract

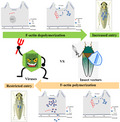

## 1 INTRODUCTION

Vector insects such as leafhoppers, planthoppers, aphids, mosquitoes and ticks play critical roles in epidemics of numerous animal and plant viruses (Gray & Gildow, 2003; Hajano et al., 2020; Hogenhout et al., 2008). Serious virus epidemics are largely attributed to high populations of an insect vector (Jia et al., 2018; Ng & Falk, 2006; Wei & Li, 2016). Three types of transmission by vector insects are known for plant viruses: nonpersistent, semipersistent and persistent (Gray & Gildow, 2003). In persistent transmission, the subject of the present study, insects can transmit the acquired virus to a plant for a long period, even for their entire life (Gray et al., 2014). Persistently transmitted viruses are known to be of two types: nonpropagative and propagative viruses. Both types must circulate in a vector body (Hogenhout et al., 2008). Generally, after ingestion during insect feeding, persistently transmitted viruses move through the alimentary canal of the insect vector, invade the gut epithelial cells, and are released into the haemolymph. Finally they infect the salivary glands and are released into the salivary ducts, where they can be delivered to plants via the saliva (Ghanim, 2014). Thus, viruses have evolved a series of strategies to interact with various components in vector cells to overcome any defence mechanisms and be retained by the insect vector through their life (He et al., 2020; Wang, Guo, et al., 2020).

Wheat dwarf virus (WDV, *Mastrevirus*, *Geminiviridae*) and barley yellow dwarf virus‐GAV (BYDV‐GAV, *Luteovirus*, *Luteoviridae*) have caused serious yield losses of cereal crops all over the world (Liu et al., 2020; Miller & Rasochova, 1997). WDV is mainly transmitted by *Psammotettix alienus* and BYDV‐GAV by *Schizaphis graminum* and *Sitobion avenae* in a persistently transmitted manner (Miller & Rasochova, 1997; Wang, Wu, et al., 2019). Previous studies found that cubam receptor‐mediated clathrin‐dependent endocytosis is important for the transport of the begomovirus tomato yellow leaf curl virus across the whitefly midgut barrier (Pan et al., 2017; Zhao et al., 2020), while the mastrevirus maize streak virus (MSV) may use a nonclathrin‐mediated endocytic pathway, possibly lipid‐raft‐mediated endocytosis, to enter gut cells (Ammar et al., 2009), suggesting that persistently transmitted viruses can use different pathways to enter these cells. Our previous study showed that ADP‐ribosylation factor 1 (ARF1) is involved in the spread of WDV from the gut to haemolymph (Wang, Liu, et al., 2019), but the mechanisms involved in WDV entry into the midgut cells and retention in the vector insect for its lifetime have not been elucidated completely.

The host cytoskeleton, consisting mostly of microtubules, microfilaments and intermediate filaments, has important roles for many viruses in completing their life cycle (Wen et al., 2021). Dynamic reorganization and rearrangement of the actin cytoskeleton is associated with various cellular processes ranging from cell motility and the maintenance of cell shape to cell division (Jimenez‐Baranda et al., 2007; Pollard & Cooper, 2009). Actin filaments are also important in maintaining cell morphology and inhibiting the invasion of pathogens (Lv et al., 2019). In addition, cortical actin below the plasma membrane is the first obstacle encountered by viruses upon infection. Actin, a ubiquitously expressed, highly conserved protein, exists in two forms: globular monomeric (G‐actin) and filamentous polymeric (F‐actin) (Dominguez & Holmes, 2011; Park et al., 2020). The actin cytoskeleton is modulated by the balance of G‐actin and F‐actin caused by actin‐associated proteins such as formin, profilin, and actin‐depolymerizing factor (ADF)/cofilin (Fu et al., 2014; Wioland et al., 2017). Among these proteins, ADF/cofilin is a common regulator of actin dynamics and is responsible for remodelling F‐actin‐based cytoskeletal structures, such as filopodia, stress fibres, and cortical actin networks by binding and severing F‐actins (Wang, Song, et al., 2020; Zheng et al., 2016). Moreover, ADF/cofilin is inactivated by phosphorylation at serine‐3, which prevents its association with actin (Arber et al., 1998). Many viruses have been reported to take advantage of insect cofilin to regulate the dynamics of F‐actin during infection to promote virion entry into cells (Nie et al., 2021). For example, entry of porcine hemagglutinating encephalomyelitis virus (PHEV) into N2a cells induces a biphasic remodelling of the actin cytoskeleton, which is regulated by cofilin (Lv et al., 2019). Human immunodeficiency virus (HIV) activates cofilin through chemokine receptor signalling to mediate entry into resting CD4^+^ T cells (Santos et al., 2014; Yoder et al., 2008). While actin cytoskeleton remodelling has been reported to be involved in host infection for many animal‐infecting viruses (Radtke et al., 2006; Xiang et al., 2012), this has so far not been demonstrated for virus acquisition and persistence in insect vectors.

Here, we report evidence that a DNA virus, WDV, induces F‐actin depolymerization in midgut cells of the vector insects during the early acquisition access period (AAP). Once the virus enters the midgut cells, the virus coat protein (CP) can bind directly with ADF to inhibit its function, resulting in F‐actin polymerization, to support viral persistence in the vector. Similarly, we found that an RNA virus, BYDV‐GAV, also induces F‐actin depolymerization and then polymerization in the gut cells of the vector aphid. This mechanism may allow persistently transmitted viruses to enter and be retained in the vector insects their entire lifespan.

## 2 RESULTS

### 2.1 WDV acquisition induces biphasic F‐actin dynamics in midgut cells

**FIGURE 1:**
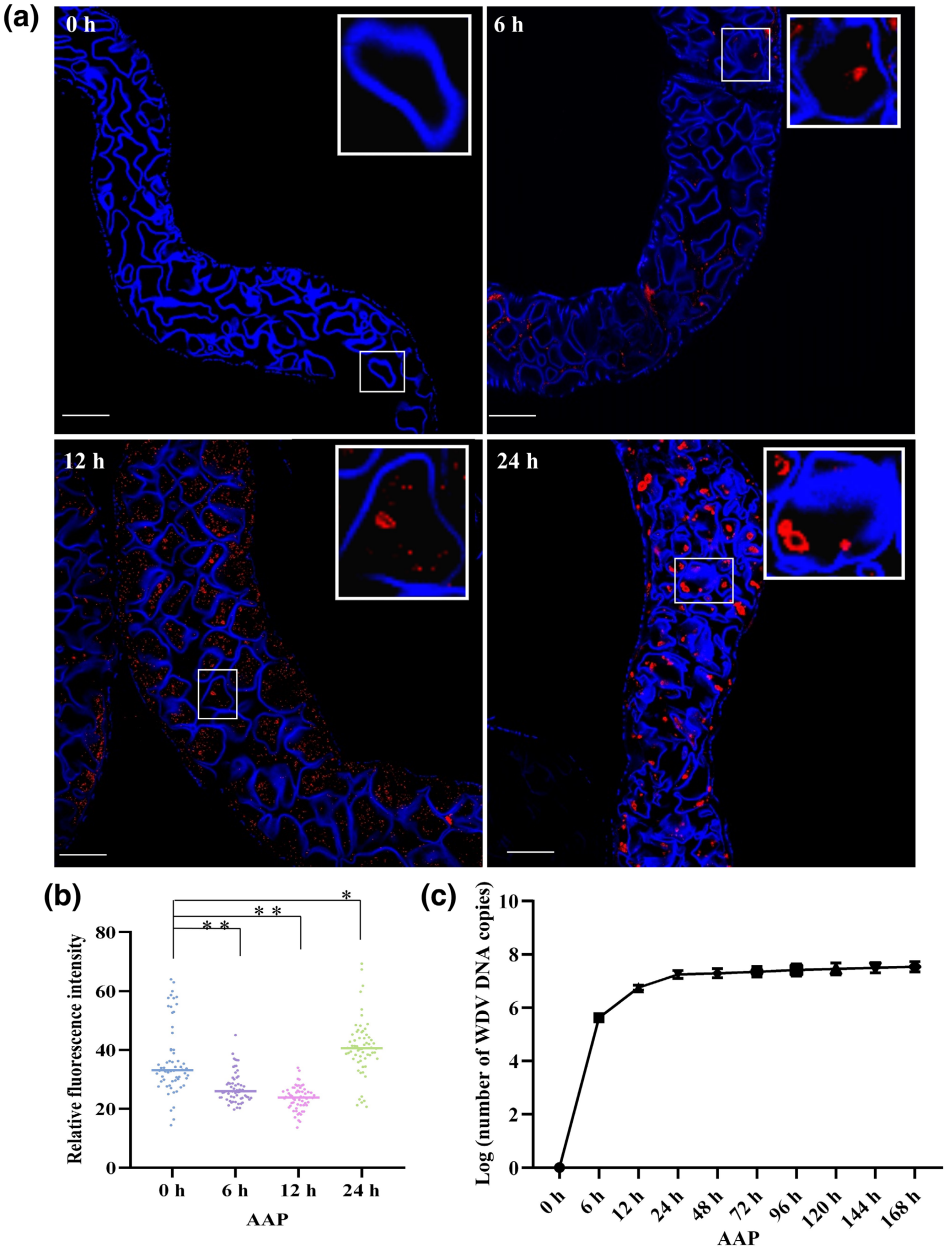
Wheat dwarf virus (WDV) entry induces F‐actin depolymerization or polymerization in gut cells of *Psammotettix alienus* at different acquisition access periods (AAPs). (a) Laser scanning confocal microscopy images showing temporal reorganization of F‐actin and distribution of WDV in gut cells (*n* = 25, three repetitions) after feeding on WDV‐infected wheat plants for a 6‐h, 12‐h or 24‐h AAP. Guts were excised, fixed, permeabilized, incubated with antibody for the coat protein (CP) of WDV (red), and stained with phalloidin (blue). Scale bars, 50 μm. (b) Fluorescence of F‐actin in gut cells after various durations of WDV acquisition to quantify F‐actin reorganization. Fluorescence was analysed using ImageJ. *p < 0.05, **p < 0.01. Each dot represents one gut sample. Horizontal lines represent the mean. (c) WDV accumulation level in leafhoppers fed on WDV‐infected plants after different AAPs.

The leafhoppers were allowed to feed on WDV‐infected wheat plants for 6, 12 or 24 h, and then the F‐actin structures in midgut cells were observed with a laser scanning confocal microscope (LSCM). In the midgut cells of nonviruliferous leafhoppers, the microfilaments were observed to form a fine homogeneous network with occasional small F‐actin aggregates in the cytoplasm near the plasma membrane (Figure 1a). At a 6‐h virus AAP, a few signals (red) of CP were observed in epithelial cells of the midgut of viruliferous leafhoppers, and microfilaments formed a discrete network with few F‐actin aggregates, which demonstrated that F‐actin had begun to depolymerize in most cells in contrast to those in the nonviruliferous leafhopper. By the 12‐h AAP, the CP signals had increased in the epithelial cells and actin filaments continued to depolymerize. At the 24‐h AAP, the CP signals were observed in the entire alimentary canal, and many actin patches had formed in the epithelial cells, suggesting that actin filaments were reassembling (Figure 1a). When we quantified the F‐actin in midgut cells using ImageJ, total F‐actin in the midgut cells of viruliferous leafhoppers was lower at an early stage of acquisition (6 to 12‐h AAP) and then higher at the 24‐h AAP compared to that in the midgut cells of nonviruliferous leafhoppers (Figure 1b). Taken together, our results suggest that WDV induced F‐actin depolymerization and then polymerization in the gut cells of its vector leafhoppers.

### 2.2 WDV accumulation level in leafhoppers fed on WDV‐infected plants at different AAPs

The number of WDV DNA copies in midgut cells was 4.2 × 10^5^ at the 6‐h AAP and reached approximately 5.7 × 10^6^ by the 12‐h AAP and 1.8 × 10^7^ by the 24‐h AAP (Figure 1c), indicating that the leafhoppers acquired virus rapidly at the early AAPs. With the extension of AAPs, the viral accumulation level in midgut cells increased slowly and was estimated at 3 × 10^7^ at 7 days, suggesting that virus acquisition by the leafhopper reached a plateau after the first 24h AAP (Figure 1c).

### 2.3 F‐actin depolymerization is involved in WDV acquisition

**FIGURE 2:**
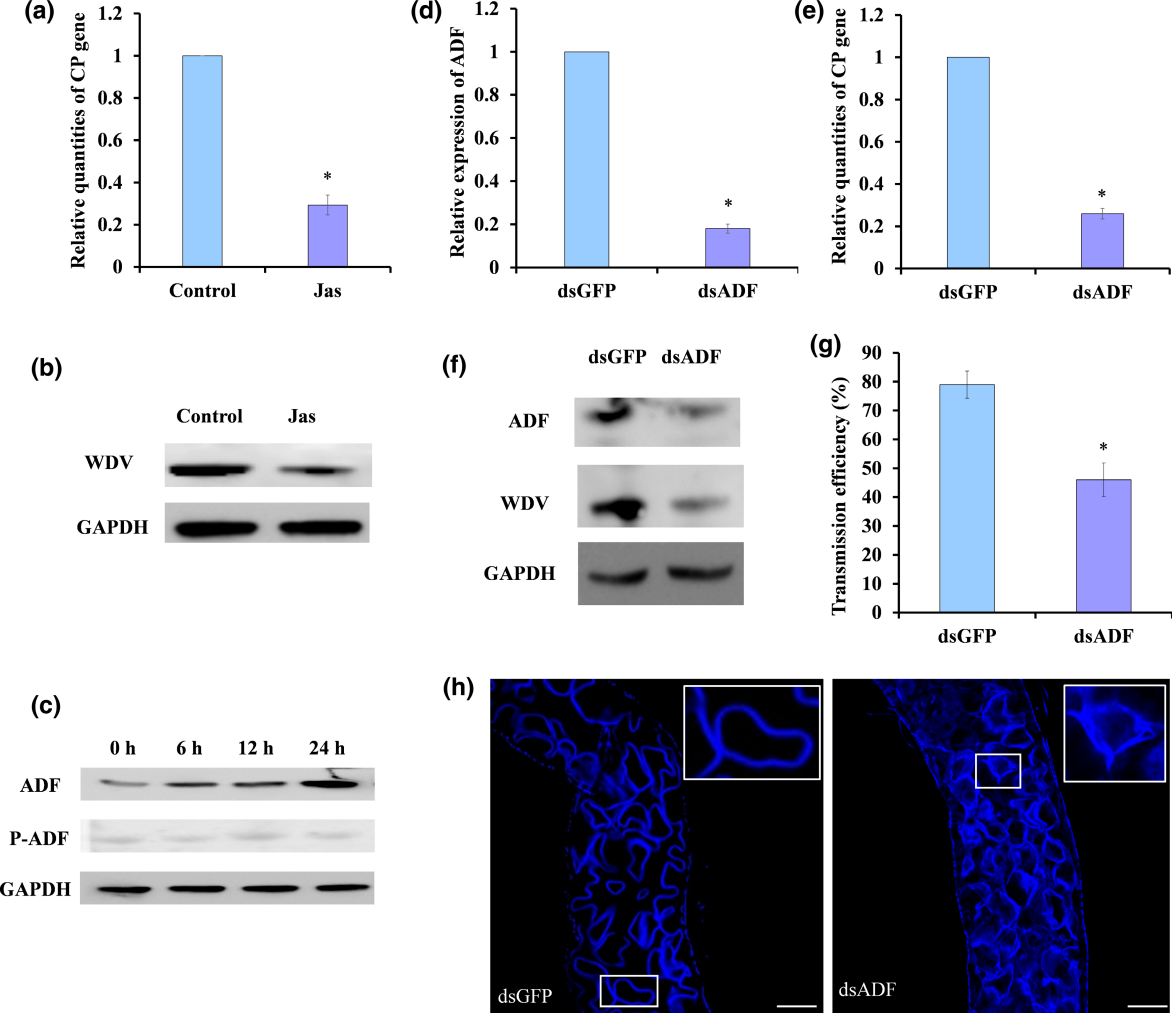
F‐actin depolymerization is essential for wheat dwarf virus (WDV) entry into gut cells. (a,b) The DNA and protein levels of coat protein (CP) in gut cells of jasplakinolide (Jas)‐treated leafhoppers as determined by quantitative PCR (qPCR) (a) and western blot (b). Leafhoppers were allowed to feed on a diet containing Jas or control diet for 12 h and then allowed a 24‐h acquisition access period (AAP) on WDV‐infected wheat plants. (c) Western blot analysis of the protein level of ADF and phosphorylated ADF (P‐ADF) in gut cells of nonviruliferous and viruliferous leafhoppers with different AAPs. P‐ADF was identified in immunoprecipitated ADF by an anti‐phosphoserine antibody. (d) Reverse transcription (RT)‐qPCR results showing reduced *ADF* transcript levels in gut cells after injection with ds*ADF*. (e) qPCR results showing that WDV *CP* levels in gut cells were reduced after injection with ds*ADF*. For qPCR and RT‐qPCR results, data are means from three separate experiments; error bars are standard deviations. *p < 0.05. (f) Western blot of ADF and CP levels in gut cells. Nonviruliferous leafhoppers that had been injected with either ds*ADF* or ds*GFP* were fed on WDV‐infected wheat for a 24‐h AAP and collected, and the gut was excised. The GAPDH protein was used as a control for the western blot assay. (g) Virus transmission efficiency by ds*ADF*‐injected leafhoppers decreased significantly compared with ds*GFP*‐injected controls. Data are means from three separate experiments; error bars are ± *SD*. *p < 0.05. (h) Laser scanning confocal microscopy images showing the F‐actin structure in gut cells from leafhoppers after injection with ds*GFP* (left) or ds*ADF* (right). Bar is 50 μm.

After leafhoppers had been fed on jasplakinolide (Jas), F‐actin in the gut cells was found to be polymerized (Figure S1). Leafhoppers were then allowed to feed on a diet containing Jas or without Jas (control) and then placed on WDV‐infected wheat plants for a 24‐h AAP. The viral DNA accumulation level in the midgut was quantified using quantitative PCR (qPCR), and the results showed that Jas treatment decreased the relative quantities of *CP* in the midgut cells, suggesting F‐actin polymerization decreases virus entry into cells (Figure 2a). Western blots also showed significantly less virus in the midgut of Jas‐treated leafhoppers than in the control leafhoppers (Figures 2b and S3a,c). Together, these results suggest that the F‐actin depolymerization is needed for WDV entry into epithelial cells.

### 2.4 WDV acquisition induces ADF up‐regulation in the midgut cells

The full‐length *ADF* (GenBank accession MW770744) was amplified from total leafhopper RNA using reverse transcription (RT)‐PCR and the nucleotide sequence was determined by Sanger sequencing. The amplified gene contains a 447‐bp open reading frame (ORF), which encodes a predicted 148‐amino acid (aa) protein, which was predicted to be a member of the ADF gelsolin superfamily (Figure S2a) and does not contain a transmembrane spanner (Figure S2b) or signal peptide (Figure S2c). We used RT‐qPCR to analyse relative transcript levels of *ADF* in different tissues of nonviruliferous leafhoppers and found higher expression in the gut than in the other tissues (Figure S2d). After the insects fed on WDV‐infected wheat plants for 6, 12 and 24 h, *ADF* expression in the gut was higher than in nonviruliferous leafhoppers (Figures 2c and S3f–h). However, this up‐regulation of *ADF* expression did not increase the level of phosphorylated ADF (P‐ADF) as determined by western blot analysis (Figures 2c and S3g,i).

### 2.5 Reducing *ADF* expression by RNA interference inhibited WDV entry into gut cells and decreased WDV transmission efficiency

After microinjection with ds*ADF* or ds*GFP* (green fluorescent protein), the leafhoppers in the two treatment groups appeared to be healthy and not different from the uninjected controls, and both had survival rates of >85% (Figures S4 and S5). Third‐instar nymphs injected with either ds*ADF* or ds*GFP* were allowed to feed on WDV‐infected plants for 24 h, and then RNA/DNA was extracted from excised guts to estimate mRNA transcript levels for *ADF* and the DNA levels of *CP*. *ADF* transcript levels and the relative quantities of *CP* decreased by about 82% and 74%, respectively, in the midgut of ds*ADF*‐injected insects compared with ds*GFP*‐injected insects (Figure 2d,e). LSCM images show that F‐actin of midgut cells was polymerized after *ADF* expression was suppressed (Figure 2h), suggesting that knockdown of *ADF* expression induced the polymerization of F‐actin and impeded the entry of WDV into the midgut cells. Western blot assays also showed that the CP level in the midgut cells was lower than in the insects injected with ds*GFP* (Figures 2f and S3b,d,e). LSCM observations showed that the midgut from 50 ds*ADF*‐injected leafhoppers had less intense ADF fluorescence (green) and WDV fluorescence (red) compared with the controls (Figure S6). Mean transmission efficiency by insects injected with ds*ADF* was 46% (average of 50%, 48% and 39%), compared to 79% (average of 83%, 80% and 74%) after ds*GFP* injection (Figure 2g). Overall, these results demonstrate that ADF‐driven F‐actin depolymerization is essential for WDV entry into gut cells of leafhoppers.

### 2.6 CP and ADF interacted in vitro and in vivo

**FIGURE 3:**
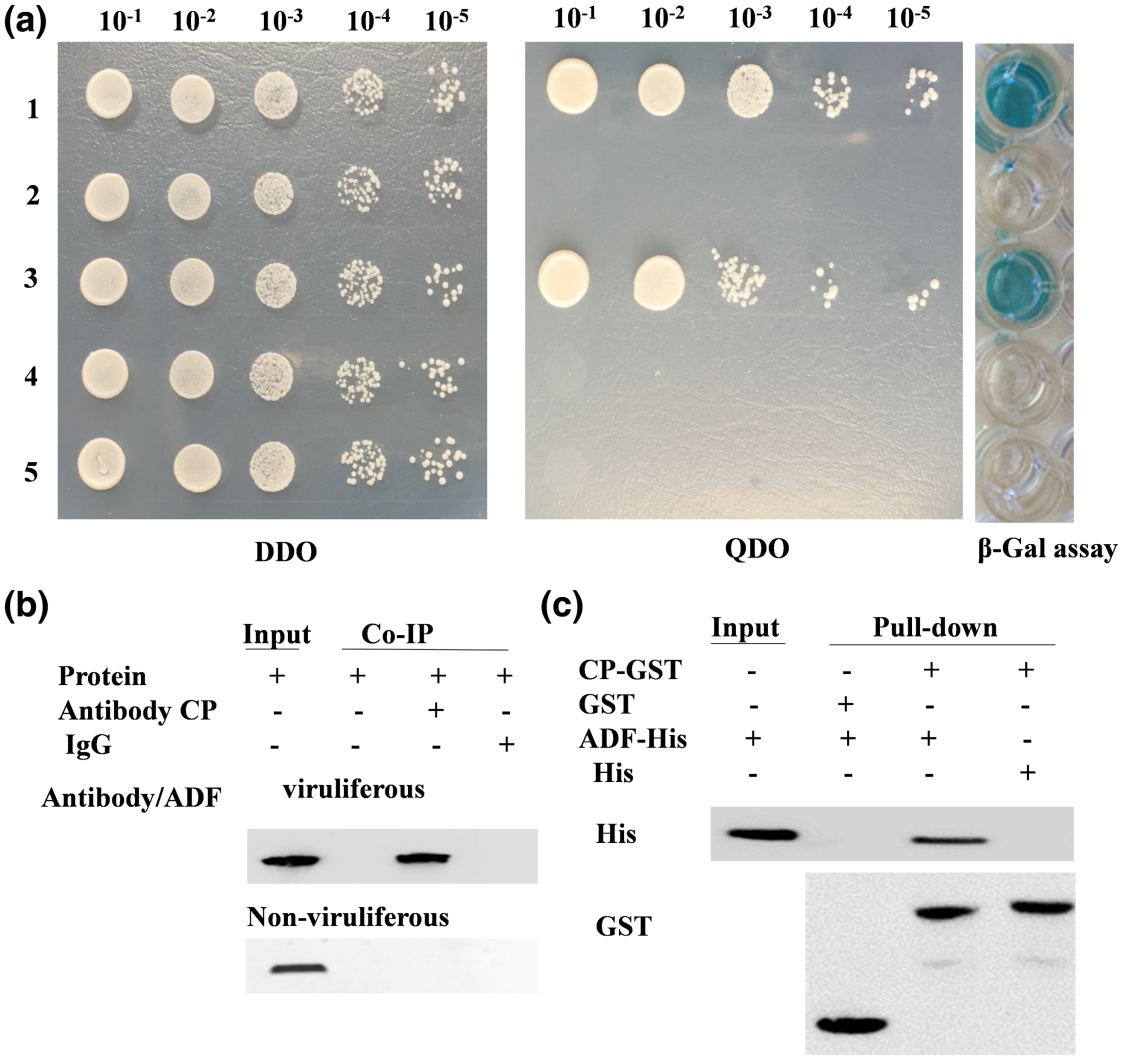
Analysis of interaction between wheat dwarf virus (WDV) coat protein (CP) and ADF in vivo and in vitro. (a) Confirmation of the interaction between CP and ADF by yeast two‐hybrid assay. Yeast strain NMY51 was cotransformed with the indicated plasmid pairs and spotted onto selective double dropout medium (DDO) and select quadruple dropout medium (QDO) in a 10‐fold dilution series; clones grown on DDO were selected for the β‐galactosidase assay. Plasmid pairs: 1, positive control, pDHB1‐largeT/pDSL‐p53; 2, negative control, pDHB1‐largeT/pPR3‐N; 3, pDHB1‐CP/pPR3‐N‐ADF; 4, pDHB1‐CP/pPR3‐N; 5, pDHB1/pPR3‐N‐ADF. (b) Confirmation of the interaction of CP and ADF by coimmunoprecipitation (Co‐IP) assay. Protein from viruliferous and nonviruliferous leafhoppers was extracted and then incubated with anti‐CP antibody and protein A/G agarose beads for immunoprecipitation. Western blots were probed with anti‐ADF antibodies. (c) In vitro interaction between glutathione S‐transferase (GST)‐tagged CP with His‐tagged ADF and detection by anti‐GST and anti‐His antibodies.

In the yeast two‐hybrid assay, the pPR3N‐ADF and pDHB1‐CP clones grew well on the quadruple dropout selective medium and turned blue in the β‐galactosidase assay, whereas no clones or blue colour developed in the controls (pPR3N‐ADF/pDHB1 and pPR3N/pDHB1‐CP), suggesting that ADF interacts with CP in yeast (Figure 3a). Similarly, in the coimmunoprecipitation assay, the anti‐CP antibody coimmunoprecipitated the ADF only in the viruliferous leafhoppers, whereas protein G‐Sepharose did not (Figure 3b). Our pulldown assay also confirmed that ADF bound to glutathione S‐transferase (GST)‐fused CP, but not to GST (Figure 3c). Taken together, CP can bind ADF in vitro and in vivo.

### 2.7 The interaction between ADF and CP influences actin distribution and structure in *Spodoptera frugiperda* 9 cells

**FIGURE 4:**
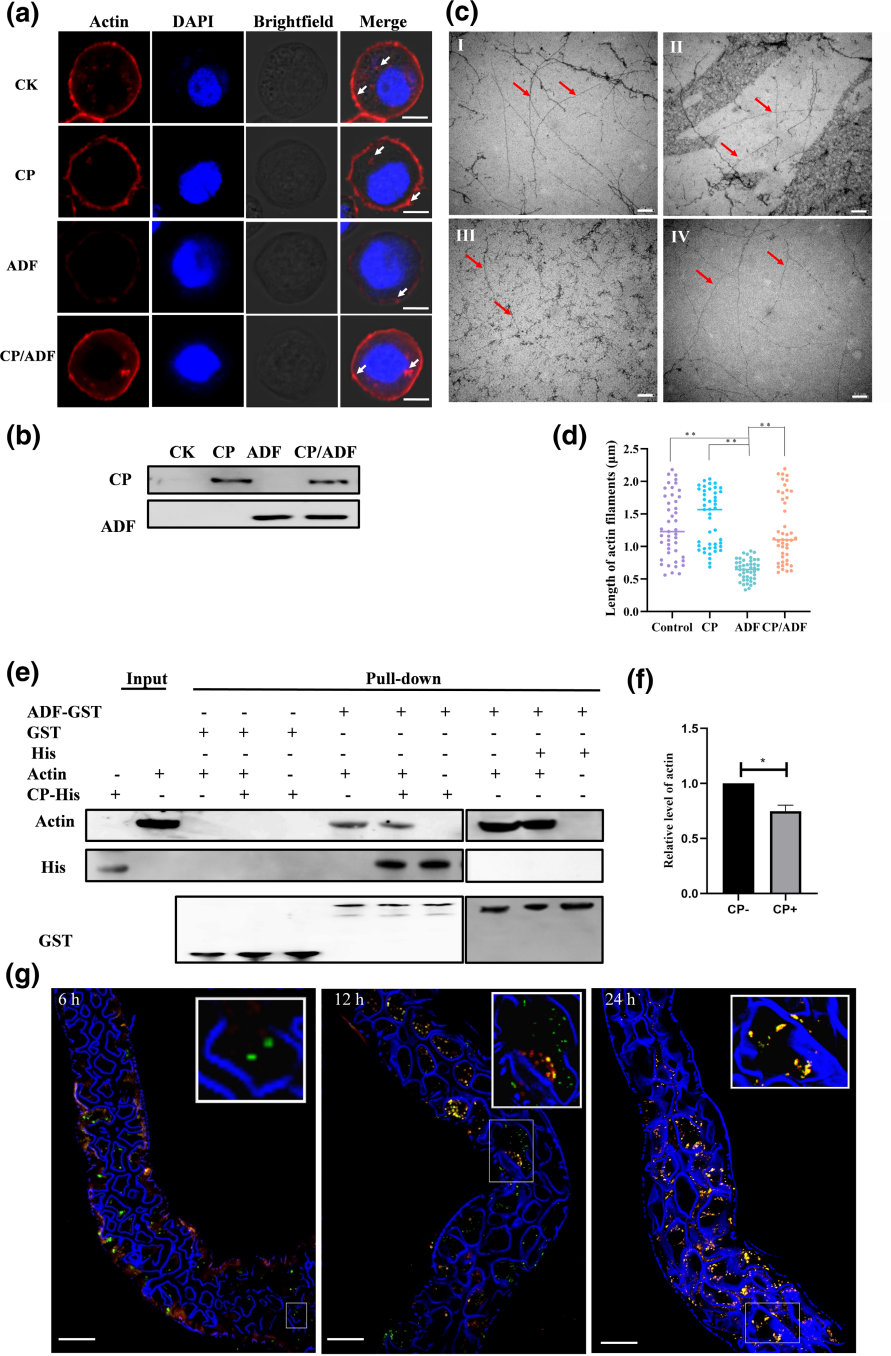
Coat protein (CP) inhibits F‐actin depolymerization via interaction with ADF. (a) Formation of F‐actin in Sf9 cells. Sf9 cells were transfected with bacmid that expressed ADF, CP, ADF and CP together, or with lipofectamine only (CK). At 48 h posttransfection, cells were fixed, permeabilized, and incubated with phalloidin and 4′,6‐diamidino‐2‐phenylindole (DAPI), followed by observation with a laser scanning confocal microscope (LSCM). White arrows: F‐actin aggregates. Scale bars: 5 μm. (b) The expression of CP (upper panel) and ADF (lower panel) in Sf9 cells was confirmed using western blot assays. (c) Micrographs to show the effect of ADF and CP on the F‐actin structure. F‐actin was incubated with buffer (I), CP (II), ADF (III), or CP and ADF (IV), negatively stained with uranyl acetate, and observed with an electron microscope. Red arrows indicate the F‐actin. Actin alone in buffer showed long filaments. Only short filaments were observed in the presence of ADF, while long filaments were observed in the presence of ADF and CP. Scale bars: 200 nm. (d) Length of actin filaments in vivo (*n* = 15, three repetitions). Each dot represents one actin filament. (e) Wheat dwarf virus (WDV) CP outcompetes ADP‐G‐actin for binding ADF. Recombinantly expressed ADF‐GST was bound to GST Sepharose as a bait and then incubated with recombinantly expressed with CP, ADP‐actin, or CP and ADP‐actin together. (f) Densitometry analysis of the bands from (e). (g) LSCM images of ADF and CP in gut cells after a 6‐, 12‐, or 24‐h acquisition access period. The inset in the top right of each image is a detail of the boxed area. Excised midguts of *Psammotettix alienus* leafhoppers were incubated with antibodies against ADF labelled with DyLight 488 (green) and antibodies against WDV CP that had been labelled with Cy3 (red).

Cultured *S. frugiperda* 9 (Sf9) cells were transfected with a bacmid that expresses ADF, CP, or ADF and CP together in parallel with mock‐transfected cells. In the mock‐transfected cells, actin filaments were distributed along the interior of the plasma membrane, and some F‐actin aggregates were formed in the cytoplasm (Figure 4a, CK). In transfected Sf9 cells that expressed ADF, F‐actin was broken down and fewer F‐actin aggregates were present in the cytoplasm than in the mock‐transfected cells (Figure 4a, ADF), demonstrating that ADF promoted depolymerization of actin filaments. When ADF and CP were coexpressed, some F‐actin aggregates were present, as in the mock‐transfected cells (Figure 4a, CP/ADF), indicating that binding of CP and ADF inhibited F‐actin depolymerization driven by ADF. Our western blot analysis confirmed that the CP and ADF were expressed in the variously transfected cells (Figure 4b). Taken together, CP inhibits F‐actin depolymerization driven by ADF in Sf9 cells.

### 2.8 CP inhibits F‐actin depolymerization driven by ADF in vitro

After polymerization in F‐buffer for 30 min at room temperature, long filaments of rabbit skeletal muscle actin were observed by electron microscopy (Figure 4cI,d). The actin filaments did not change when CP was added (Figure 4cII,d). However, after ADF was added, only a few long actin filaments were present, whereas short filaments were frequently observed (Figure 4cIII,d), indicating that F‐actin had depolymerized. Interestingly, long actin filaments were seen when CP and ADF were added together, suggesting that CP inhibited the function of ADF (Figure 4cIV,d). Also, the pulldown assay showed that ADF bound to adenosine diphosphate (ADP)‐G‐actin. However, when CP was present, binding of ADP‐G‐actin to ADF was reduced (Figure 4e,f). Taken together, CP competed with actin to bind ADF and then blocked actin filament disassembly.

### 2.9 CP inhibits F‐actin depolymerization driven by ADF in vivo

The alimentary canal of the leafhopper was excised and incubated with anti‐ADF antibody labelled with DyLight 488 (green) and anti‐WDV antibody labelled with DyLight Cy3 (red) for LSCM visualization of the distribution of WDV CP and ADF in the alimentary canal over time. After the 6‐h AAP, WDV CP was observed in only a few epithelial cells of the midgut, and very few virions colocalized with ADF in the cell cytoplasm (Figures 4f and S7). WDV CP levels were increased in epithelial cells, and somewhat more WDV CP had colocalized with ADF after the 12‐h AAP (Figures 4f and S7). Almost all WDV CP had colocalized with ADF in the epithelial cells by the 24‐h AAP, corresponding to the time when F‐actin was polymerizing (Figures 4f and S7). Taken together, CP inhibits F‐actin depolymerization driven by ADF in gut cells of leafhoppers.

### 2.10 BYDV‐GAV entry also induces F‐actin depolymerization or polymerization in gut cells of *S. graminum* at different AAPs

**FIGURE 5:**
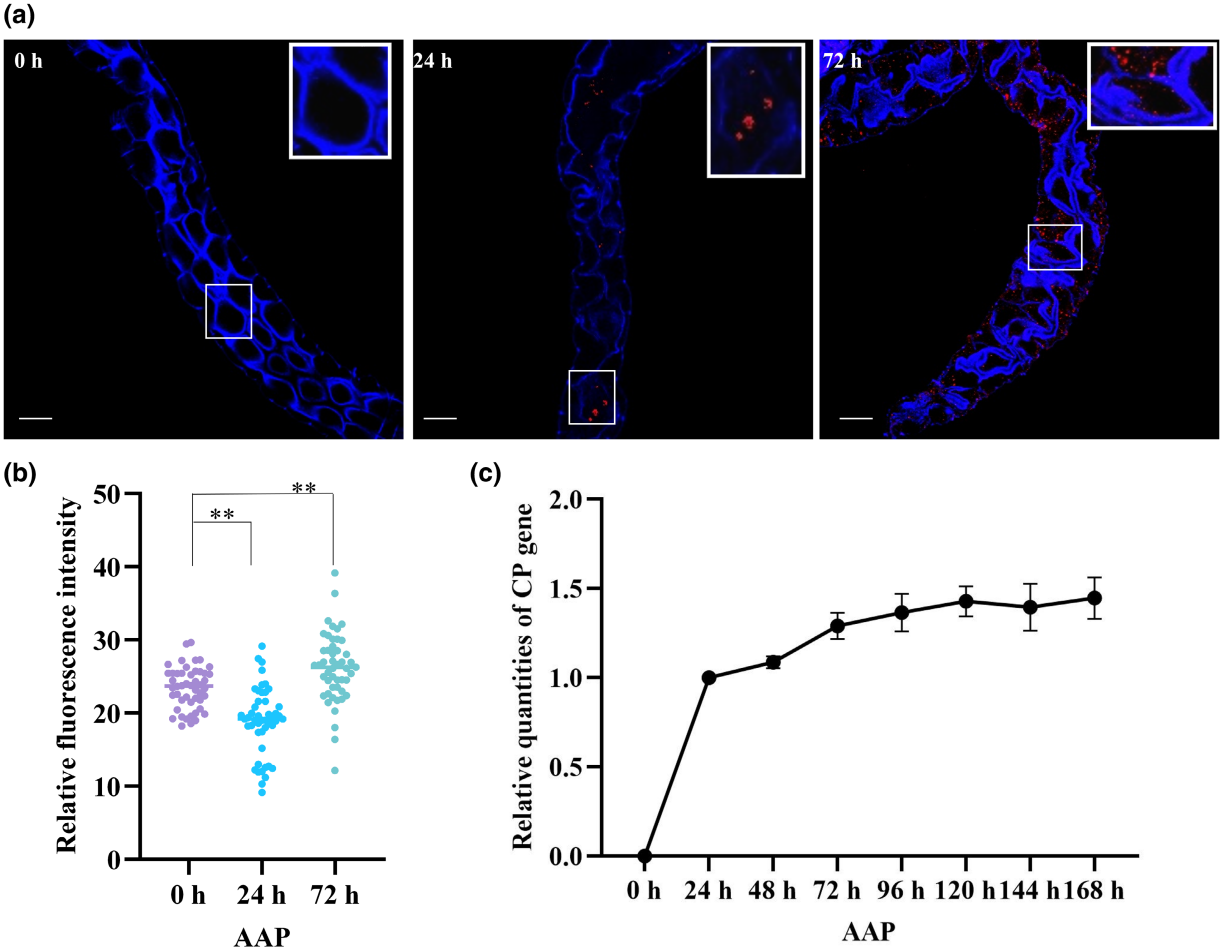
Barley yellow dwarf virus‐GAV (BYDV‐GAV) entry induces F‐actin depolymerization or polymerization in gut cells of *Schizaphis graminum* at different acquisition access periods (AAPs). (a) Laser scanning confocal microscopy images showing reorganization of F‐actin in gut cells (*n* = 20, three repetitions) after feeding on BYDV‐GAV‐infected plants for a 24‐h or 72‐h AAP. Guts were excised, fixed, permeabilized, incubated with antibody for BYDV‐GAV (red), and stained with phalloidin (blue). Scale bars, 20 μm. (b) Fluorescence of F‐actin in gut cells after various durations of BYDV‐GAV acquisition to quantify F‐actin reorganization. Fluorescence was analysed using ImageJ. **p < 0.01. Each dot represents one gut sample. Horizontal lines represent the mean. (c) The BYDV‐GAV coat protein (*CP*) accumulation level in *S. graminum* fed on BYDV‐GAV‐infected plants after different AAPs was detected by reverse transcription‐quantitative PCR.

To explore whether F‐actin dynamics play a conserved role in persistent circulative viruses, we conducted similar tests with another persistently transmitted virus, BYDV‐GAV, which is transmitted by *S. graminum*. Immunofluorescence assays showed that F‐actin depolymerized in most cells of *S. graminum* at the 24‐h AAP, in contrast to those in nonviruliferous aphids, while F‐actin polymerized at the 72‐h AAP compared with those in the nonviruliferous aphids (Figure 5a). We also quantified the F‐actin in midgut cells using ImageJ, and the total F‐actin level in the midgut cells of viruliferous aphids was lower at the 24‐h AAP and then higher at the 72‐h AAP compared to that in the midgut cells of nonviruliferous aphids (Figure 5b). The accumulation level of BYDV‐GAV in *S. graminum* increased gradually from 24 h to 72 h, while it tended to stabilize after 72 h (Figure 5c). These data indicate that the F‐actin dynamics may play a similar role in other persistently transmitted viruses.

### 2.11 WDV cannot change the structure of F‐actin in gut cells of *S. graminum*

*S. graminum* is an important economic pest that is a serious threat to wheat, but it does not transmit WDV. Consistently, WDV cannot be detected in aphids after feeding on WDV‐infected wheat seedling for 1–7 days (Figure S8a). Moreover, WDV did not influence the structure of F‐actin in gut cells of aphids (Figure S8b). These findings indicate that WDV cannot change the structure of F‐actin in gut cells of nonvector aphids.

### 2.12 Transmission efficiency of WDV by *P. alienus* after different AAPs

Leafhoppers can transmit WDV to new healthy plants for 32–36 days by a 6‐h AAP on WDV‐infected plants and for 36–40 days by a 12‐h AAP, a 24‐h AAP, or a 48‐h AAP. Transmission efficiencies of leafhopper were significantly increased at 28 days and 32 days by the 12‐h AAP compared to the 6‐h AAP (Table S2). Transmission efficiencies of leafhoppers were significantly increased at 20 days and 24 days by the 24‐h AAP compared to the 12‐h AAP (Table S2). There was no significant difference in transmission efficiency between the 24‐h AAP and the 48‐h AAP (Table S2). Thus, the transmission efficiency increased significantly with the extension of the AAP within the first 24‐h AAP and then increased slightly after the 24‐h AAP.

## 3 DISCUSSION

Viruses usually use the host cell cytoskeleton for their infection cycle (Smith & Enquist, 2002). Although some studies have focused on the function of proteins in the cytoskeleton that are involved in virus traffic in vector insects (Mao et al., 2017), very little research has been conducted on the pathways of plant virus entry into, and persistence in, the midgut cells of vector insects. Here we provided evidence that WDV acquisition induces F‐actin depolymerization in the midgut epithelial cells early in the acquisition stage, after a 6‐h AAP and a 12‐h AAP, suggesting that F‐actin depolymerization might be involved in virus entry into gut cells. When viruses bind with a cellular attachment factor or receptor, they often induce F‐actin rearrangement to overcome a physical barrier such as the plasma membrane and the actin cytoskeleton (Yoder et al., 2008; Zheng et al., 2014). HSV‐1 entry requires a two‐phase process of rapid actin assembly and disassembly (Xiang et al., 2012; Zheng et al., 2014). During early infection, viral binding induces F‐actin polymerization to induce receptor clustering and initiate the entry process; subsequent viral penetration leads to the depolymerization of existing F‐actin, which facilitates virus entry into the cell (Zheng et al., 2014). In the case of PHEV entry into N2a cells, F‐actin rapidly polymerizes within 5 min postinoculation (mpi), and by 20 mpi it starts to depolymerize, a state essential for PHEV invasion because disruption of either actin depolymerization or polymerization reduces PHEV entry into the host cells (Lv et al., 2019). Similar to our results, binding of the HIV envelope to the chemokine coreceptor CXCR4 in resting lymphocytes triggers signal transduction and leads to cortical actin depolymerization to help HIV penetrate CD4^+^ T cells because inhibition of the CXCR4 signal pathway by pertussis toxin or inhibition of F‐actin depolymerization by Jas decreases the virus titre in T cells (Yoder et al., 2008). These previous findings and our results suggest that viruses often trigger actin rearrangement processes when they infect a host cell; however, the process of rearrangement of the actin cytoskeleton induced varies depending on the type of virus.

The ADF/cofilin family has been characterized as a group of actin‐binding proteins critical for controlling the assembly of actin within cells (Pollard, 2016; Wioland et al., 2017). ADF/cofilin can bind actin monomers and depolymerize actin filaments by severing filaments to reorganize the F‐actin (Bamburg & Wiggan, 2002; Dai et al., 2013). Generally, host entry by a virus requires active ADF/cofilin to cleave the F‐actin that accumulates on the interior of the cell membrane and to modulate the cytoskeleton (Lv et al., 2019; Zheng et al., 2014). During HIV‐1 infection, binding of glycoprotein 120 (gp120) to CXCR4 triggers Gai‐dependent signal transduction that leads to cofilin activation for the virus to penetrate T cells (Yoder et al., 2008). Here we found ADF was up‐regulated at the transcript and protein levels in the gut cells of leafhoppers after WDV acquisition, probably because the virus bound to a specific receptor and then induced one signalling pathway to regulate the expression of *ADF* or reduce protein degradation. At the same time, ADF induced F‐actin depolymerization, and WDV quickly entered leafhopper gut cells, suggesting ADF‐driven F‐actin depolymerization is essential for WDV entry into midgut cells. Previous studies have focused on actin rearrangement induced by animal viruses and the dependence on cofilin for penetration into host cells (Yoder et al., 2008; Zheng et al., 2014). We provide direct evidence that this plant virus uses ADF to depolymerize F‐actin and enter gut cells of the insect vector.

The epithelial cells of the midgut and the filter chamber of *Cicadulina mbila* have also been demonstrated to act as a reservoir for MSV; the acquired virus is then retained in an infective form for the lifespan of this leafhopper vector (Ammar et al., 2009). Because *C. mbila* can transmit MSV to new healthy plants for 35 days after a 3‐h AAP on MSV‐infected maize plants, a short AAP is apparently sufficient for abundant virions to enter, be retained in the gut cells or filter chamber, and thus be persistently transmitted (Reynaud & Peterschmitt, 1992). In our study, after a 6‐h AAP and a 12‐h AAP, WDV uses ADF to induce actin depolymerization and quickly enter gut cells of the leafhopper vector, indicating that F‐actin depolymerization is essential for WDV entry into these cells. We further found that after a 6‐h AAP, the leafhoppers can transmit WDV to new plants for 32–36 days, probably because many virions entered and were retained in gut epithelial cells after the short AAPs. Thus, we infer that F‐actin depolymerization is an effective way for large amounts of WDV to enter gut cells and be persistently transmitted.

Interestingly, we found that actin polymerization is induced later in the acquisition stage, after a 24‐h AAP. Direct interaction between CP and ADF in the yeast two‐hybrid assay, the pulldown assay, and the coimmunoprecipitation assay further demonstrated potential binding of ADF and CP in vitro and in vivo. We then explored whether CP altered the function of ADF because previous studies have found that virus proteins can inhibit cofilin‐driven disassembly of F‐actin (Nawaz‐ul‐Rehman et al., 2016). For example, expression of p33 or p92 replication proteins of tomato bushy stunt virus (TBSV) in yeast is known to inhibit cofilin‐driven disassembly of actin filaments and lead to the formation of a large actin filament patch, which promotes formation of the viral replicase complex and replication of TBSV (Nawaz‐ul‐Rehman et al., 2016). In the present study, ADF could depolymerize actin filaments while CP inhibited ADF‐driven disassembly of F‐actin in vitro and in Sf9 cells, suggesting WDV can induce F‐actin polymerization by inhibiting the function of ADF. Moreover, we found that WDV CP colocalized with ADF after a 24‐h AAP, corresponding to the time when F‐actin was polymerized, and virus accumulation reached a plateau (24‐ to 168‐h AAP). Thus, after a 24‐h AAP, WDV can hijack ADF and disturb actin reorganization to inhibit virus entry into the gut cells of leafhoppers, and extending the AAP after 24 h led to slightly higher transmission efficiencies. In general, cofilin/ADF activity is regulated by phosphorylation, which prevents the association of cofilin with actin (Arber et al., 1998), whereas dephosphorylation by phosphatases activates cofilin (Ambach et al., 2000). Recently, swine fever virus was reported to regulate the activity of cofilin to infect porcine kidney (PK‐15) cells by inducing its phosphorylation and dephosphorylation through the epidermal growth factor receptor–phosphatidylinositol 3‐kinase–mitogen‐activated protein kinase–Ras homologue family member A–Ras‐related C3 botulinum toxin substrate 1–cell division control protein 42 homologue (EGFR–PI3K–MAPK–RhoA–Rac1–Cdc42) signalling pathway (Cheng et al., 2021). The lentiviral Nef protein, a key pathogenicity factor, inactivates cofilin molecules to inhibit cell mobility via its interaction with the host Pak2 kinase that phosphorylates cofilin (Stolp et al., 2009). We also examined the expression levels of P‐ADF in the leafhopper guts after WDV acquisition, but it did not change significantly. According to our pulldown assay, CP bound ADF in competition with ADP‐actin. A previous study also found that the interaction of p33 replication protein of TBSV with cofilin‐1p inhibits cofilin‐1p interaction with ADP‐actin and blocks actin filament disassembly and recycling of monomeric actin to form new actin filaments in the yeast cells (Nawaz‐ul‐Rehman et al., 2016). Our results provide evidence that the binding of CP and ADF in the midgut epithelial cells would disturb F‐actin depolymerization by inhibiting ADF interaction with ADP‐actin.

At first glance, it does not make sense that WDV would inhibit the function of ADF because ADF‐regulated F‐actin depolymerization is needed for virus entry into gut cells of the vector. For persistently transmitted viruses, large amounts of virions enter gut cells of the vector within the first several hours of the vector's access to the infected host, and the virus accumulates slowly or the virus accumulation level plateaus as the AAP is extended (Wu et al., 2014; Zeidan & Czosnek, 1991). As aphids feed longer on infected oat plants, the titre of BYDV‐PAV and WDV‐GPV in *Rhopalosiphum padi* gradually increases from 12 h to 60 h or 72 h, but tends to stabilize after 60 h or 72 h (Wu et al., 2014). Another study also found that whiteflies could not acquire more than 6 × 10^9^ virus genome copies of TYLCV, suggesting the existence of factors that control the number of virions present in an insect (Zeidan & Czosnek, 1991). It seems that high viral accumulation levels would be deleterious to the survival of insect vectors. The vectors in turn have evolved a series of strategies to control virus accumulation in the body (Wang et al., 2016; Xu et al., 2015). Several begomoviruses, and particularly TYLCV, have also been shown to negatively impact the fitness of *Bemisia tabaci* (Ghanim, 2014; Rosen et al., 2015). For example, a 48‐h AAP on TYLCV‐infected tomato plants decreased the lifespan of whiteflies that were initially reared on eggplant, a TYLCV nonhost, by 5 to 7 days and decreased fecundity by 25% to 50% compared to nonviruliferous whiteflies (Ghanim, 2014). As a strategy to control virus accumulation to enable their survival, the vector whiteflies can activate the autophagy pathway to degrade the TYLCV (CP) and genomic DNA as a direct defence mechanism against the retention of TYLCV (Wang et al., 2016). Tomato yellow leaf curl China virus also suppressed whitefly immune responses by down‐regulating the expression of genes involved in Toll‐like signalling and MAPK pathways to enable their spread in the vector whitefly (Luan et al., 2011).

**FIGURE 6:**
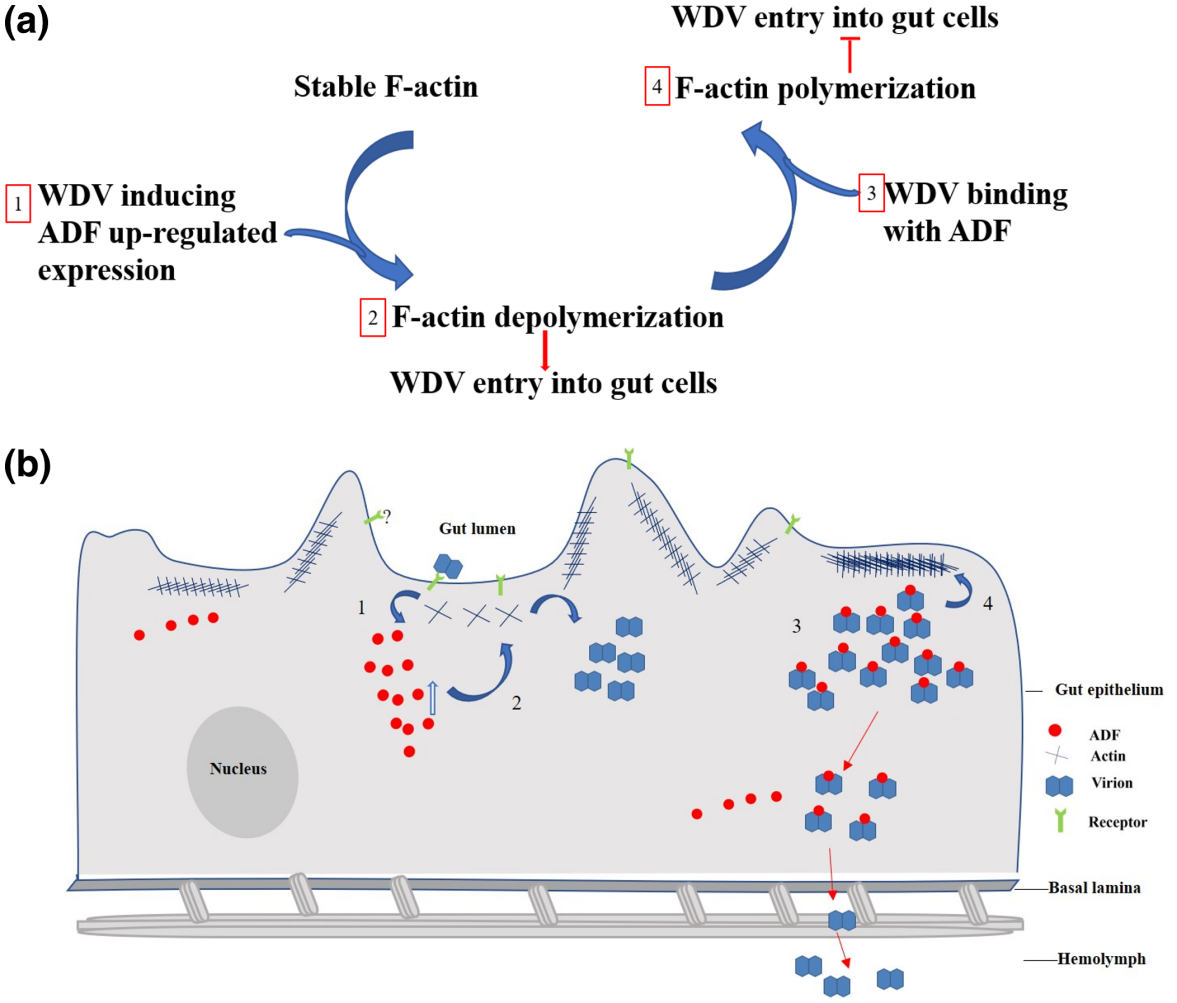
Model of wheat dwarf virus (WDV) entry into midgut epithelial cells of leafhoppers via regulating actin dynamics. (1–3) At an early acquisition stage, WDV induces the up‐regulated expression of ADF at the transcript and protein levels, which relaxes F‐actin inside the cell near the cell membrane to facilitate WDV entry into gut cells. (4) After a longer period of acquisition, coat protein (CP) competitively binds ADF with ADP‐actin, blocks F‐actin depolymerization, and controls virus entry into the cells for persistent transmission. Thus, biphasic dynamics of F‐actin mediated by the ADF–CP interaction may play an important role in controlling virus entry and the virus accumulation level required for persistent transmission while maintaining the health of the vector.

Our study revealed a new mechanism in which plant viruses hijack ADF to disturb F‐actin rearrangement and thus control the virus accumulation level in the insect vector. Thus, insects may control virus accumulation by directly inhibiting virion entry into gut cells or by degrading virions already in the gut cells. However, different to some begomoviruses, which can negatively affect their whitefly vector, the negative impact of WDV acquisition on leafhoppers has not been reported (Wang et al., 2016). According to our long‐term observations, viruliferous leafhoppers develop well when reared on healthy plants. Thus, we propose WDV and its vector coevolved mutually beneficial interactions to regulate the virus threshold and allow the virus to persist in the insect vector (Figure 6). Interestingly, we found that BYDV‐GAV also induces F‐actin depolymerization of gut cells in aphids at an early acquisition stage and polymerization at a later acquisition stage. Thus, this model may also be applicable to other persistently transmitted viruses with insect vectors.

The host actin cytoskeleton plays an essential role in virion budding and release from cells. Measles virus matrix protein associates with actin filaments (F‐actin) to regulate virion assembly and budding (Wakimoto et al., 2013). Rabies virus inactivates cofilin to facilitate viral budding and release (Zan et al., 2016). ARF6, a member of the ARF family, is also an important regulator of cytoskeletal reorganization (D'Souza‐Schorey et al., 1997; D'Souza‐Schorey & Chavrier, 2006). For example, the GTPase‐defective mutant of ARF6 (Q67L) remodels the actin cytoskeleton by inducing actin polymerization at the Chinese hamster ovary cell periphery (D'Souza‐Schorey et al., 1997). Our previous study found that ARF1 helps virus spread from the gut to haemolymph (Wang, Liu, et al., 2019). Therefore, further efforts are warranted to determine whether ARFs of leafhopper function as important regulators of cytoskeletal reorganization in response to WDV spread from the gut to the haemolymph.

To summarize, WDV induces F‐actin depolymerization after a short acquisition time by the vector because F‐actin depolymerization is essential for virus entry into gut cells of the leafhopper. Moreover, up‐regulation of *ADF* leads to relaxation of F‐actin near the cell membrane of the gut cells, thus permitting entry of large amounts of WDV. However, after a prolonged acquisition period of WDV, F‐actin polymerization is induced in the leafhopper. We further found that CP interacts with ADF and inhibits ADF‐driven F‐actin depolymerization in vitro in Sf9 cells and in gut cells because the CP outcompetes ADP‐G‐actin to bind ADF and thus blocks actin filament disassembly, suggesting that the binding between CP and ADF confers a disadvantage to WDV when invading the gut cells. Thus, biphasic dynamics of F‐actin, mediated by the interaction between ADF and CP in midgut cells, may play an important role in controlling the virus accumulation level required for their persistent transmission while maintaining the health of the vector. The fact that F‐actin also depolymerizes and then polymerizes in gut cells of *S. graminum* after acquisition of BYDV‐GAV suggests that persistently transmitted viruses might use a similar strategy to enable their transmission.

## 4 EXPERIMENTAL PROCEDURES

### 4.1 Insect, virus and antibodies

Leafhoppers (*P. alienus*) were originally collected from Hancheng city, Shaanxi Province. Nonviruliferous leafhoppers were reared on healthy wheat seedlings (*Triticum aestivum* 'Yangmai 12') in an insect‐proof containment chamber with 16 h light/8 h dark at 22°C and transferred to fresh seedlings every 25 days to ensure sufficient nutrition.

WDV‐infected wheat plants have been maintained in the laboratory for many years (Wang et al., 2014). About 10–15 wheat seeds were planted in a plastic pot containing nutrient soil in a growth chamber (22°C/20°C with a 16 h light/8 h dark cycle). When wheat seedlings were 2–3 cm high, more than 30 WDV‐viruliferous leafhoppers were placed on the seedlings for 2 days. Then, the insects were removed, and plants were grown for 3 weeks in the growth chamber. After 3 weeks, PCR was used to test the wheat plants for WDV when symptoms appeared.

Laboratory isolates of BYDV‐GAV have been maintained on oat plants (*Avena sativa* ‘Coast‐Black’) in our laboratory since the 1990s. Nonviruliferous aphids (*S. graminum*) were reared on wheat seedlings, under controlled conditions at 18–23°C (Wu et al., 2014).

The rabbit anti‐WDV CP antibody was produced by our laboratory (Wang et al., 2014). The mouse anti‐ADF monoclonal antibody was prepared by Abmart. The anti‐phosphoserine antibody was purchased from Abcam. The rabbit anti‐BYDV‐MAV antibody was purchased from Agdia. Horseradish peroxidase (HRP)‐conjugated goat anti‐mouse IgG, HRP‐conjugated goat anti‐rabbit IgG, mouse monoclonal anti‐GAPDH, and mouse monoclonal anti‐His tag were procured from Protech. Alexa Fluor 488 goat anti‐mouse IgG, Cy3 goat anti‐rabbit IgG, and Alexa Fluor 633 phalloidin were obtained from Invitrogen and DAPI was purchased from Thermo Scientific.

### 4.2 WDV acquisition by leafhopper vectors from WDV‐infected wheat plants in different AAPs

Leafhoppers were maintained on WDV‐infected wheat plants for feeding and virus acquisition. At each sampling date after the different AAPs (6, 12, 24, 48, 72, 96, 120, 144 and 168 h), 10 insects were collected and frozen at −80°C. DNA was extracted with the Wizard Genomic DNA Purification Kit (Promega) and tested with specific primers for WDV by PCR after the last sampling. The standard plasmids pQE80L‐CP were used in 10‐fold serial dilutions to generate standard curves to determine the assay efficiency and quantify the viral target in the unknown samples (Wang et al., 2014).

### 4.3 Virus acquisition assay after feeding on jasplakinolide (Jas)

For assessing the role of F‐actin in virus acquisition, leafhoppers were fed on Jas, a chemical that induces the polymerization and stabilization of actin filaments, in 15% sucrose solution through Parafilm for 12 h and then transferred to a WDV‐infected plant for virus acquisition. After 24 h of feeding, leafhoppers were removed from the infected plant. Leafhoppers that were fed on 15% sucrose solution and then placed on a WDV‐infected plant served as a control. The DNA of midgut from 30 leafhoppers was extracted to quantify *CP* gene expression using qPCR. Total protein was extracted from the midgut from 50 leafhoppers to quantify CP using a western blot assay.

### 4.4 Cloning, sequencing, and analysis of *ADF*

*ADF* was cloned and sequenced as described. The sequence was used to search for reference sequences using BLASTX against the nonredundant (nr) NCBI database. Transmembrane structures were predicted using the online server TMHMM server v. 2.0 (http://www.cbs.dtu.dk/services/TMHMM/), and SignalP was used to predict signal peptide sites (http://www.cbs.dtu.dk/services/SignalP/).

### 4.5 RT‐qPCR analysis of expression of *ADF* in different leafhopper tissues

To study the distribution of ADF in different tissues of leafhopper, we collected internal organs, including the gut, salivary glands, haemolymph, ovary, testes, and the remaining carcass, from nonviruliferous leafhoppers as described (Wang et al., 2019). The total RNA was extracted and the *ADF* mRNA transcript level was quantified by RT‐qPCR.

### 4.6 dsRNA preparation

dsRNA of *ADF* and *GFP* was synthesized using the T7 Ribomax Express RNAi system (Promega) according to the manufacturer's instructions. Briefly, the *ADF*/*GFP* sequence for dsRNA synthesis was amplified by PCR with primers containing the T7 RNA polymerase promoter (Table S1), and the purified PCR products were incubated with enzyme (contained in the kit) at 37°C for 30 min to generate RNA transcripts.

The synthesized dsRNA was then precipitated with isopropanol and resuspended in nuclease‐free water. The concentration of dsRNA was quantified with a NanoDrop 2000 (Thermo Scientific) and the integrity of dsRNA was confirmed by 1% agarose gel electrophoresis.

### 4.7 Virus acquisition, spread and transmission after RNA interference

To assess the role of ADF in virus acquisition in the vectors, third‐instar leafhoppers were injected with 23 nl *ADF* dsRNA (3 μg/μl) or with *GFP* dsRNA (3 μg/μl) as a control using an Auto‐Nanoliter Injector (Drummond) and allowed 48 h on healthy wheat seedlings. Then they were transferred to WDV‐infected wheat plants for a 24‐h AAP. In total 30 leafhoppers treated in this way were collected and the midgut was excised. Furthermore, total RNA from the midgut from 30 leafhoppers was extracted to estimate *ADF* transcript levels by RT‐qPCR, and DNA of the midgut from 30 leafhoppers was extracted to quantify the *CP* gene using qPCR. Total protein was extracted from the midgut of 50 leafhoppers to quantify ADF and CP using a western blot assay (Zhang et al., 2021). To assess the transmission efficiency, 50 leafhoppers were transferred to wheat seedlings (one insect per plant) for a 24‐h inoculation access period, and the seedlings were then grown in the greenhouse. After 21 days, each wheat plant was observed for virus symptoms and tested by PCR as described above. The experiments were performed three times.

### 4.8 Yeast two‐hybrid assay

The *CP* and *ADF* gene sequences were cloned into the vectors pDHB1 and pPR3‐N, respectively. The yeast two‐hybrid assay was performed using a DUALhunter starter kit (Dualsystems Biotech) according to the manufacturer's protocol. Briefly, yeast two‐hybrid assays were carried out by cotransformation of *Saccharomyces cerevisiae* NMY51 with *CP* and *ADF* using the lithium acetate method with single‐stranded DNA as the carrier (Gietz & Woods, 2002). The mixture was plated on selective double dropout medium (SD/−Leu/−Trp), and clones were replated on selective quadruple dropout medium (SD/−Ade/−His/−Leu/−Trp) with a dilution series. The strength of the protein–protein interaction was confirmed in a β‐galactosidase assay using the HTX high‐throughput β‐galactosidase assay kit (Dualsystems Biotech).

### 4.9 Protein expression and GST pulldown assay

The *CP* and *ADF* genes were cloned into pGEX‐6p‐1 (pGEX‐6p‐1‐CP‐GST) and pQE80L (pQE80L‐CP‐His) vectors or pGEX‐6p‐1 (pGEX‐6p‐1‐ADF‐GST) and pCOLD (pCOLD‐ADF‐His) vectors using the primers in Table S1. *Escherichia coli* containing an expression plasmid was incubated at 37°C until the optical density at 600 nm of the cells reached 0.6–0.8. After a 4‐h induction with 0.4 mM isopropyl‐d‐thiogalactoside at 37°C, cells were pelleted by centrifugation, and then the pellet was resuspended with phosphate‐buffered saline (PBS), broken by ultrasonication, and centrifuged for 10 min at 12,000 × *g*. The supernatant from the sonicated cells was used for a pulldown assay or protein purification. The GST pulldown assay was done using a GST Protein Interaction Pull‐Down Kit (Pierce) according to the manufacturer's protocol. In brief, the GST‐tag‐fused protein CP‐GST was bound to glutathione Sepharose beads for 3 h at 4°C. The mixtures were centrifuged for 5 min at 100 × *g* and then the supernatants were discarded. The beads were washed with PBS five times. The His‐tagged ADF protein was added to the beads and samples were incubated for 2 h at 4°C. After centrifugation and five washes with PBS, the bead‐bound proteins were detected by western blotting with anti‐His and anti‐GST antibodies. For western blot analysis, an equal amount of protein from each sample was separated by SDS‐PAGE. Proteins were transferred to nitrocellulose membranes (General Electric), which were incubated with the indicated primary antibodies. Membranes were then incubated with species‐specific HRP‐conjugated secondary antibodies. Immunoreactive bands were visualized using the Super ECL Western Blotting Detection Kit (YTHX) and a Molecular Imager ChemiDoc XRS System (Bio‐Rad). To identify the relationship among WDV, actin and ADF, recombinantly expressed ADF‐GST was bound to GST Sepharose as a bait and then incubated with recombinantly expressed CP, ADP‐actin, or CP and ADP‐actin together. The results were identified by western blot with anti‐His, anti‐GST and anti‐actin. The experiments were performed three times.

### 4.10 Coimmunoprecipitation assay

Leafhopper proteins were extracted using cell lysis buffer (Promega). WDV CP antibodies were incubated with viruliferous leafhopper protein or nonviruliferous leafhopper protein (negative control) for 4 h at 4°C. Then protein A/G‐agarose beads were added and incubated for an additional 2 h at 4°C. In addition, proteins from either viruliferous or nonviruliferous leafhoppers were incubated with protein A/G‐agarose beads directly as negative control. After washing five times with lysis buffer, immunoprecipitated proteins were boiled in loading buffer for 5 min and detected by western blot with anti‐ADF antibody. The experiments were performed three times.

### 4.11 Immunofluorescence assay

Leafhoppers were dissected in PBS and cleared twice. Guts were fixed in 4% vol/vol paraformaldehyde in PBS for 2 h at 4°C and washed twice in PBS. After that, the guts were permeabilized with 2% Triton X‐100 for 2 h at 37°C. After three washes in PBS, guts were incubated with rabbit antibody for WDV CP at a 1:100 dilution and mouse monoclonal ADF antibody at a 1:100 dilution for 2 h at 37°C. Anti‐CP binding was detected with goat anti‐rabbit labelled with Cy3 and ADF antibody binding was detected with goat anti‐mouse labelled with Alexa Fluor 488. Alexa Fluor 633 phalloidin was incubated with leafhopper guts to visualize the structure of F‐actin using an LSCM (Zeiss). Sf9 cells and guts of aphids were treated with the same protocol. All software parameters were the same for the same LSCM experiments. The data were analysed by ImageJ v. 1.52 (NIH).

### 4.12 Actin severing assay in vitro

Rabbit skeletal muscle actin (Yuanye Biotechnology) was polymerized in F‐buffer (100 mM KCl, 2 mM MgCl_2_, 1 mM ATP, 10 mM Tris, pH 7.5) for 30 min at room temperature. F‐actin was mixed with the following proteins in G‐buffer (10 mM Tris, 0.2 mM CaCl_2_, 0.2 mM ATP, 2 mM DTT, pH 7.4) for 5 min: (1) ADF (0.1 μM), (2) CP (0.1 μM), or (3) ADF and CP together. After the incubation, samples were fixed on carbon‐supported formvar‐coated grids and negatively stained with 1% wt/vol aqueous uranyl acetate. Micrographs were taken with a transmission electron microscope at an acceleration voltage of 80 kV.

### 4.13 Cell transfection

The *CP* and *ADF* genes were inserted into the vector pFAST‐T1 and pFAST‐HTB, respectively, using the primers in Table S1. The recombinant plasmids were introduced into *E. coli* DH10Bac cells for transposition into the bacmid. The recombinant bacmid was used to transfect Sf9 cells. Sf9 cells were incubated in Sf‐900 III SFM Serum‐Free medium containing 5% vol/vol newborn calf serum at 27°C. The transfection of Sf9 cells with the pFAST‐T1‐CP and pFAST‐HTB‐ADF bacmids was performed with lipofectamine LTX reagent (Invitrogen). For the six‐well transfection, 1 μg bacmid and lipofectamine were diluted in 100 μl reduced‐serum medium (Invitrogen). After 5 min of incubation, lipofectamine LTX was added to the bacmid dilution and samples were incubated for another 30 min. The transfection mixture was then added to a 6‐well tissue culture plate containing Sf9 cells. A transfection mixture without any bacmid was included as the mock‐transfected control. After 5 h, each mixture was replaced with growth medium. After 2 days, the transfected Sf9 cells were fixed, permeabilized, and subsequently incubated with phalloidin and DAPI as described above. The F‐actin structure was observed with LSCM.

### 4.14 qPCR and RT‐qPCR

Total RNA was isolated using TRIzol reagent (Invitrogen) according to the manufacturer's instructions. The RNA concentration was determined using a NanoDrop 2000C spectrophotometer (Thermo Scientific). cDNA was synthesized using the Fast Quant reverse transcription kit (Tiangen). Total DNA was extracted using the Wizard Genomic DNA isolation kit (Promega) according to the manufacturer's instructions. Viral genomic DNA was quantified using qPCR. The mRNA transcript level of *ADF* was determined using RT‐qPCR. The primers are listed in Table S1. The *RPS23e* gene was quantified in parallel as an endogenous control. qPCR was carried out using an ABI 7500 real‐time PCR system (Applied Biosystems) with SYBR Green detection (DBI Bioscience). The cycling programme for all amplifications was 94°C for 2 min, followed by 40 cycles of 94°C for 10 s and 60°C for 30 s. All samples were tested with three independent repeats and all qPCR data were calculated using the 2^−ΔΔ*C*t^ method. Differences were statistically evaluated using Student's *t* test in SPSS v. 17.0 (IBM).

### 4.15 Detection of F‐actin depolymerization or polymerization in gut cells of *S. graminum* that acquired BYDV‐GAV at different AAPs

Aphids were maintained on BYDV‐GAV‐infected wheat plants for feeding and virus acquisition. At each sampling date after the different AAPs (0, 24, 48, 72, 96, 120, 144 and 168 h), 20 insects were collected and frozen at −80°C. Total RNA was extracted to estimate the BYDV‐GAV accumulation level by RT‐qPCR. To observe the structure of F‐actin in the gut cells of nonviruliferous and viruliferous aphids, 20 aphids were collected and dissected after the 0‐, 24‐ and 72‐h AAP, respectively, for the immunofluorescence assay. All experiments were performed three times.

## AUTHOR CONTRIBUTIONS

X.W. designed the experiments and reviewed the manuscript. X.W. and H.W. performed the experiments, analysed the data and wrote the manuscript. H.W., Y.L. and W.L. and K.W. did preliminary data processing, analysis and manuscript correction. Y.L. and W.L. and K.W.: revised the manuscript. All authors read and approved the final manuscript.

## CONFLICT OF INTEREST

The authors declare that they have no competing interests.

## Supporting information


**Figure S1** LSCM images of F‐actin structure in excised gut from leafhoppers that fed for 12 h on a diet containing jasplakinolide (Jas) (a) and 15% sucrose (control) (b). Excised guts were incubated with phalloidin and then observed. Scale bars, 50 μm

**Figure S2** Characterization of actin‐depolymerizing factor (ADF). (a) ADF belongs to the ADF gelsolin superfamily. (b,c) The amino acid sequence of ADF was submitted to online servers to predict its structure. The TMHMM server v. 2.0 found no transmembrane structure for ADF (b), and the SignalP 4.1 server found no signal peptide (c). (d) Reverse transcription quantitative PCR analysis of relative expression of *ADF* in different tissues. h: haemolymph, sg: salivary glands, ov: ovaries, te: testes, ca: remainder of carcasses. Mean of three independent experiments is shown. Error bars are ± *SD*. *p* < 0.05 (one‐way analysis of variance)

**Figure S3** Supplement of Figure 2. Two replicates of Figure 2b (a). Densitometry analysis for the image bands from Figure 2b (c). Two replicates of Figure 2c (b). Densitometry analysis for the image bands from Figure 2c (d,e). Reverse transcription quantitative PCR analysis of the *ADF* transcript level in gut cells of nonviruliferous and viruliferous leafhoppers with different AAPs (f). Two replicates of Figure 2f (g). Densitometry analysis for the image bands from Figure 2f (h,i)

**Figure S4** Survival rates of leafhoppers with or without injection with ds*GFP* and ds*ADF*. At 48 h after injection with dsRNA, the number of dead leafhoppers was inspected after. In total 50 leafhoppers were tested. All experiments were performed in triplicate

**Figure S5** *ADF* transcript level and LSCM images of the F‐actin structure in the excised gut from leafhoppers with or without injection with ds*GFP*. (a) *ADF* transcript level in gut cells from leafhoppers with or without injection with ds*GFP* as determined by reverse transcription quantitative PCR. (b,c) LSCM images of the F‐actin structure in excised guts without dsRNA (b) or with ds*GFP* (c) were incubated with phalloidin (blue). Size bar, 50 μm. In total 50 leafhoppers were tested. All experiments were performed in triplicate

**Figure S6** Confocal micrographs showing different WDV accumulation levels in the gut excised from leafhoppers after injection with ds*ADF* or ds*GFP*. Excised guts after injection with ds*GFP* (a) or ds*AD*F (b) were incubated with anti‐ADF labelled with DyLight 488 (green) and anti‐WDV labelled with Cy3 (red). Size bar, 50 μm. Fluorescence of virus was analysed using ImageJ (c)

**Figure S7** The plot profile presented was used to verify the visual colocalization in Figure 5g

**Figure S8** WDV cannot change the F‐actin structure in gut cells of *Schizaphis graminum*. (a) Detection of WDV in *S. graminum* after feeding on WDV‐infected wheat seedlings for different AAPs using PCR with WDV‐specific primers. 1–4: 24‐h AAP, 5–8: 48‐h AAP, 9–12: 72‐h AAP, 13–16: 168‐h AAP. P: positive control, N: negative control. (b) LSCM images of the F‐actin structure in the excised gut from aphids after feeding on healthy wheat plants (left) and WDV‐infected wheat plants for 24 h (right)

**Table S1** Primers used in this study

**Table S2** Transmission efficiency (%) of WDV by *Psammotettix alienus* after different AAPs

## Data Availability

The data that support the findings of this study are openly available in GenBank at https://www.ncbi.nlm.nih.gov/genbank, reference number MW770744. Other data are available from the corresponding author upon request.
