# Supplementary material for: F‐actin dynamics in midgut cells enables virus persistence in vector insects
Source: Mol Plant Pathol. 2022 Sep 8;23(11):1671–85. doi: 10.1111/mpp.13260 (PMC9562576; doi:10.1111/mpp.13260)
Supplement: Supplementary file 1 — Figure S1 LSCM images of F‐actin structure in excised gut from leafhoppers that fed for 12 h on a diet containing jasplakinolide (Jas) (a) and 15% sucrose (control) (b). Excised guts were incubated with phalloidin and then observed. Scale bars, 50 μm [file MPP-23-1671-s006.docx]

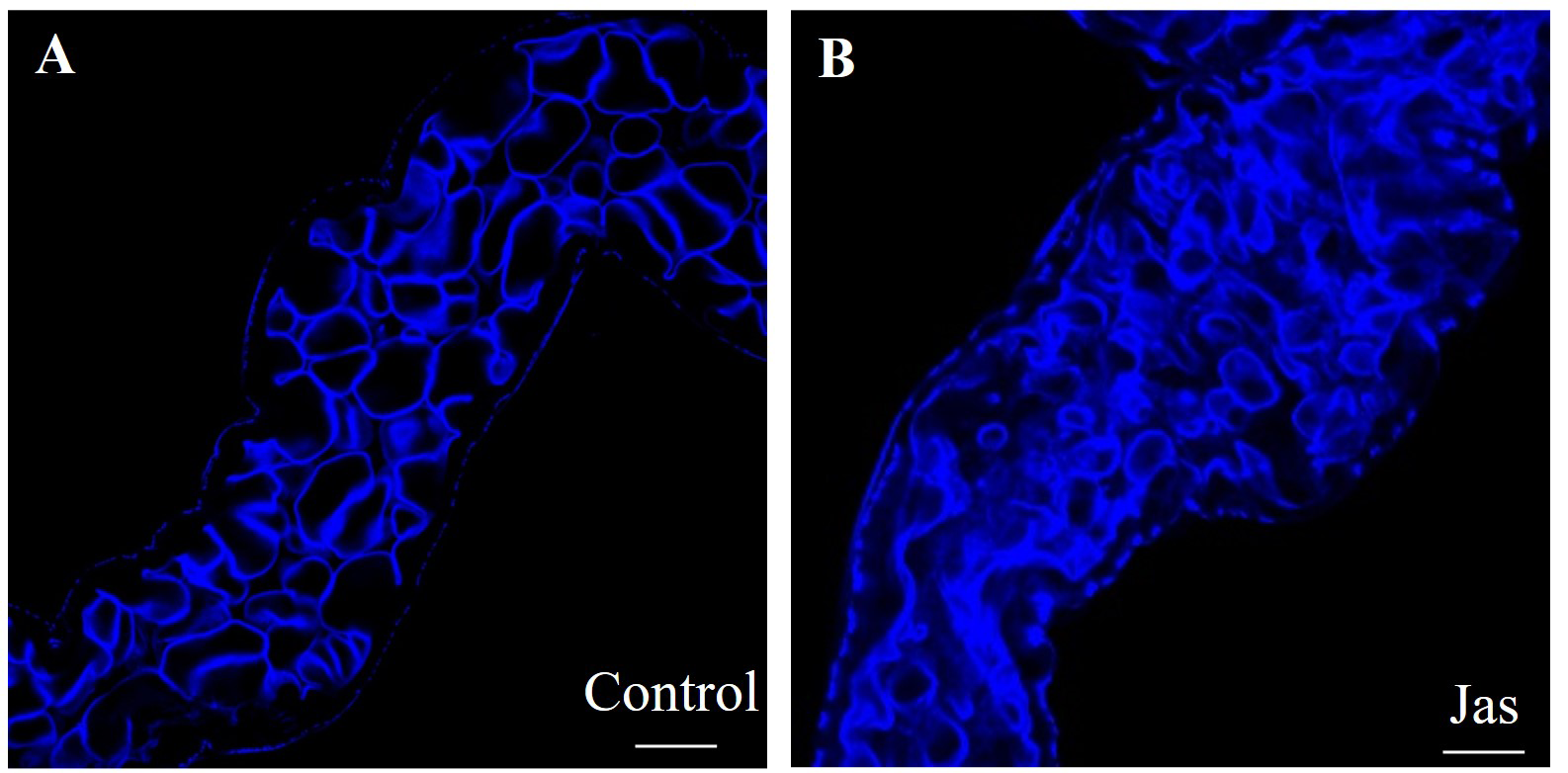


**Figure S1.** LSCM images of F-actin structure in excised gut from leafhoppers that fed for 12 h on a diet containing jasplakinolide (Jas) (A) and 15% sucrose (control) (B). Excised guts were incubated with phalloidin, then observed. Scale bars, 50 μm.
