# Supplementary material for: F‐actin dynamics in midgut cells enables virus persistence in vector insects
Source: Mol Plant Pathol. 2022 Sep 8;23(11):1671–85. doi: 10.1111/mpp.13260 (PMC9562576; doi:10.1111/mpp.13260)
Supplement: Supplementary file 2 — Figure S2 Characterization of actin‐depolymerizing factor (ADF). (a) ADF belongs to the ADF gelsolin superfamily. (b,c) The amino acid sequence of ADF was submitted to online servers to predict its structure. The TMHMM server v. 2.0 found no transmembrane structure for ADF (b), and the SignalP 4.1 server found no signal peptide (c). (d) Reverse transcription quantitative PCR analysis of relative expression of ADF in different tissues. h: haemolymph, sg: salivary glands, ov: ovaries, te: testes, ca: remainder of carcasses. Mean of three independent experiments is shown. Error bars are ± SD. p < 0.05 (one‐way analysis of variance) [file MPP-23-1671-s009.docx]

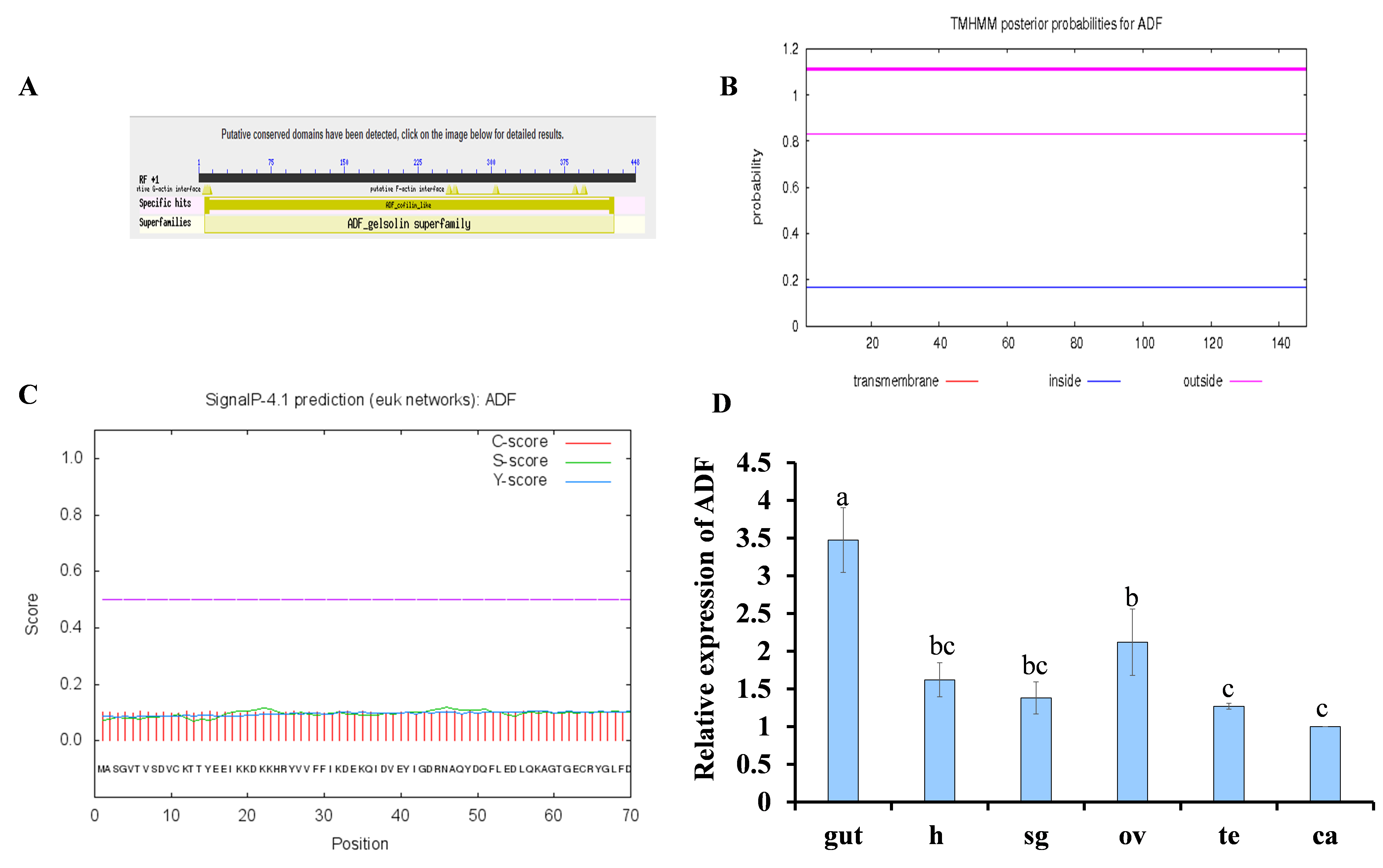


**Figure S2.** Characterization of actin-depolymerizing factor (ADF). (A) ADF belongs to the ADF gelsolin superfamily. (B and C) Amino acids in ADF were submitted to online servers to predict structure; TMHMM Server v. 2.0 found no transmembrane structure for ADF (B), and Signal P 4.1 Server found no signal peptide (C). (D) RT-qPCR analysis of relative expression of ADF in different tissues. h: hemolymph, sg: salivary glands, ov: ovaries, te: testes, ca: remainder of carcasses. Mean of three independent experiments is shown. Error bars are ± standard deviations. P<0.05 (one-way ANOVA).
