# Supplementary material for: F‐actin dynamics in midgut cells enables virus persistence in vector insects
Source: Mol Plant Pathol. 2022 Sep 8;23(11):1671–85. doi: 10.1111/mpp.13260 (PMC9562576; doi:10.1111/mpp.13260)
Supplement: Supplementary file 3 — Figure S3 Supplement of Figure 2. Two replicates of Figure 2b (a). Densitometry analysis for the image bands from Figure 2b (c). Two replicates of Figure 2c (b). Densitometry analysis for the image bands from Figure 2c (d,e). Reverse transcription quantitative PCR analysis of the ADF transcript level in gut cells of nonviruliferous and viruliferous leafhoppers with different AAPs (f). Two replicates of Figure 2f (g). Densitometry analysis for the image bands from Figure 2f (h,i) [file MPP-23-1671-s004.docx]

**
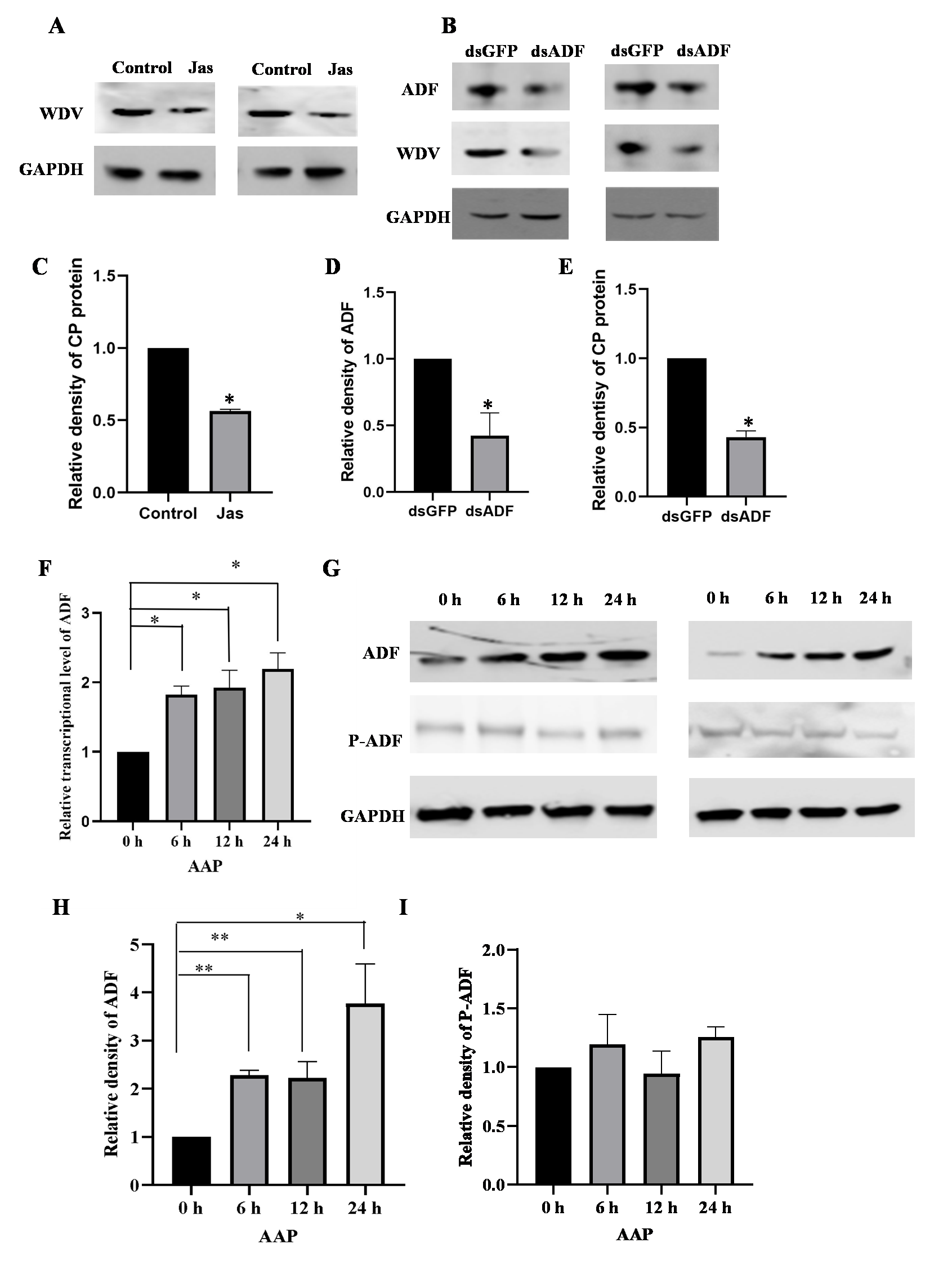
**

**Figure S3.** Supplements of the figure 2. Two replicates of figure 2B (A) Densitometry analysis for the image bands from Figure 2B (C). Two replicates of figure 2C (B). Densitometry analysis for the image bands from Figure 2C (D E). RT-qPCR analysis of ADF transcriptional levelin gut cells among nonviruliferous and viruliferous leafhopper with different AAPs (F). Two replicates of figure 2F (G). Densitometry analysis for the image bands from Figure 2F (H I).
