# Supplementary material for: F‐actin dynamics in midgut cells enables virus persistence in vector insects
Source: Mol Plant Pathol. 2022 Sep 8;23(11):1671–85. doi: 10.1111/mpp.13260 (PMC9562576; doi:10.1111/mpp.13260)
Supplement: Supplementary file 4 — Figure S4 Survival rates of leafhoppers with or without injection with dsGFP and dsADF. At 48 h after injection with dsRNA, the number of dead leafhoppers was inspected after. In total 50 leafhoppers were tested. All experiments were performed in triplicate [file MPP-23-1671-s001.docx]

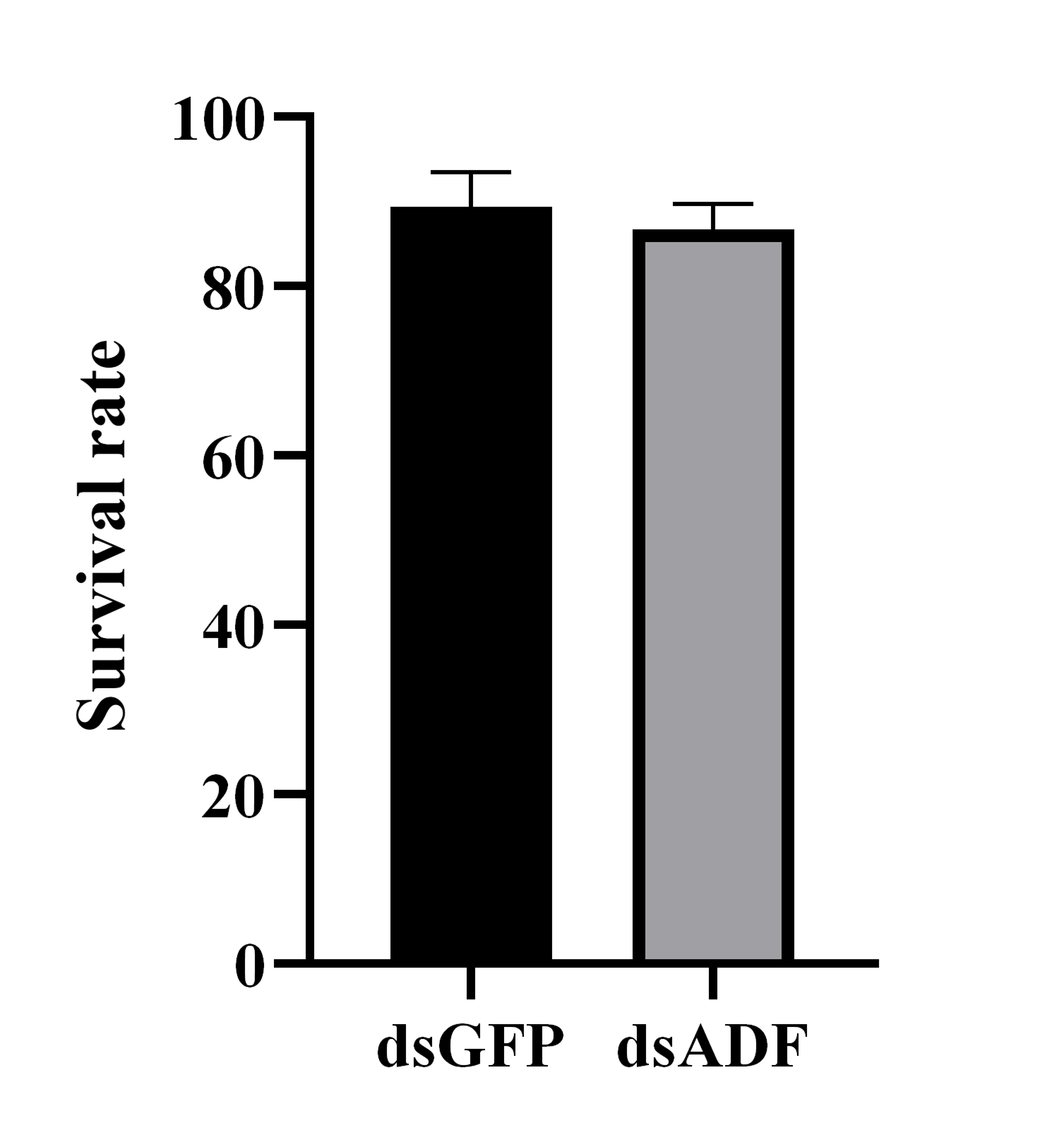


**Figure S4.** Survival rates of leafhoppers after injection with ds*GFP* and ds*ADF* not. After injection with dsRNA, the number of dead leafhoppers was inspected after 48 h. Fifty leafhoppers were tested, all experiments do three times.
