# Supplementary material for: F‐actin dynamics in midgut cells enables virus persistence in vector insects
Source: Mol Plant Pathol. 2022 Sep 8;23(11):1671–85. doi: 10.1111/mpp.13260 (PMC9562576; doi:10.1111/mpp.13260)
Supplement: Supplementary file 5 — Figure S5 ADF transcript level and LSCM images of the F‐actin structure in the excised gut from leafhoppers with or without injection with dsGFP. (a) ADF transcript level in gut cells from leafhoppers with or without injection with dsGFP as determined by reverse transcription quantitative PCR. (b,c) LSCM images of the F‐actin structure in excised guts without dsRNA (b) or with dsGFP (c) were incubated with phalloidin (blue). Size bar, 50 μm. In total 50 leafhoppers were tested. All experiments were performed in triplicate [file MPP-23-1671-s007.docx]

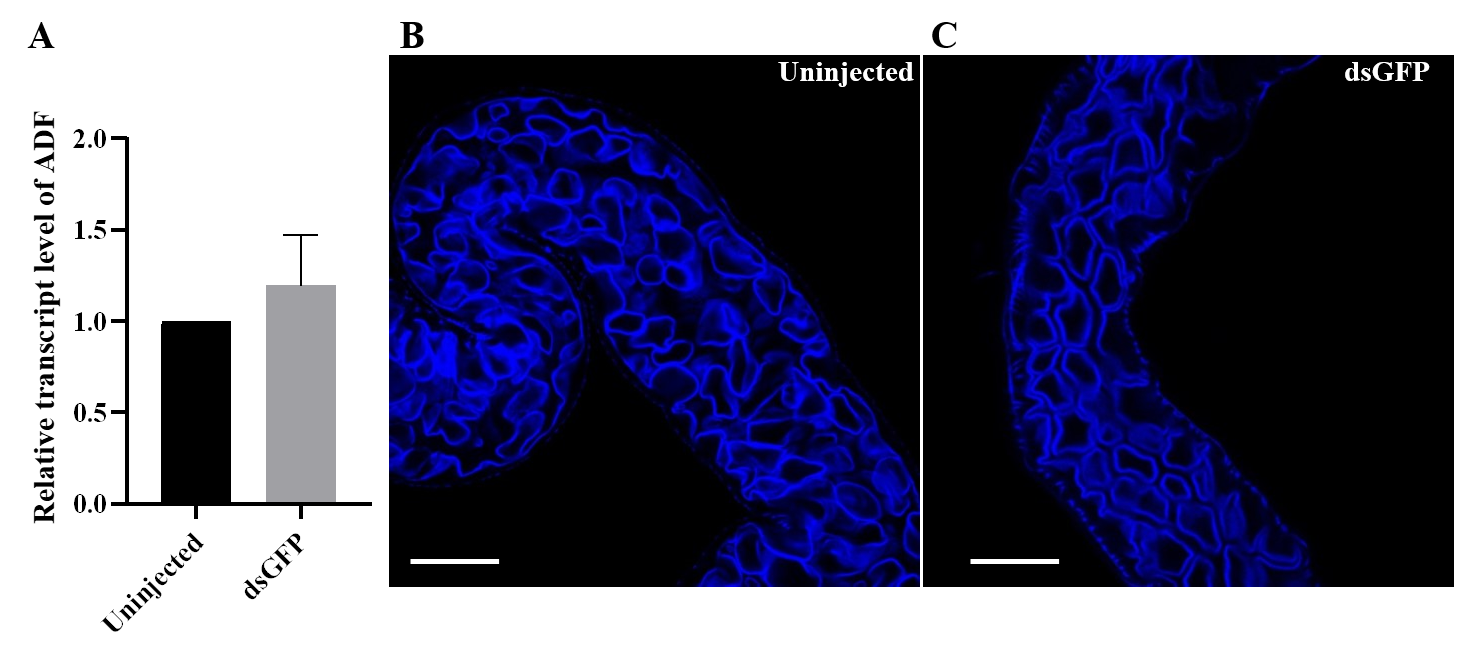


**Figure S5.** ADF transcript level and LSCM images of F-actin structure in excised gut from leafhoppers after injection with ds*GFP* or not. (A) ADF transcript level in gut cells from leafhoppers after injection with ds*GFP* or not as determined by RT-qPCR (B and C) LSCM images of F-actin structure in excised guts without dsRNA (B) or ds*GFP* (C) were incubated with phalloidin (blue). Size bar, 50 μm. Fifty leafhoppers were tested, all experiments do three times.
