# Supplementary material for: F‐actin dynamics in midgut cells enables virus persistence in vector insects
Source: Mol Plant Pathol. 2022 Sep 8;23(11):1671–85. doi: 10.1111/mpp.13260 (PMC9562576; doi:10.1111/mpp.13260)
Supplement: Supplementary file 6 — Figure S6 Confocal micrographs showing different WDV accumulation levels in the gut excised from leafhoppers after injection with dsADF or dsGFP. Excised guts after injection with dsGFP (a) or dsADF (b) were incubated with anti‐ADF labelled with DyLight 488 (green) and anti‐WDV labelled with Cy3 (red). Size bar, 50 μm. Fluorescence of virus was analysed using ImageJ (c) [file MPP-23-1671-s008.docx]

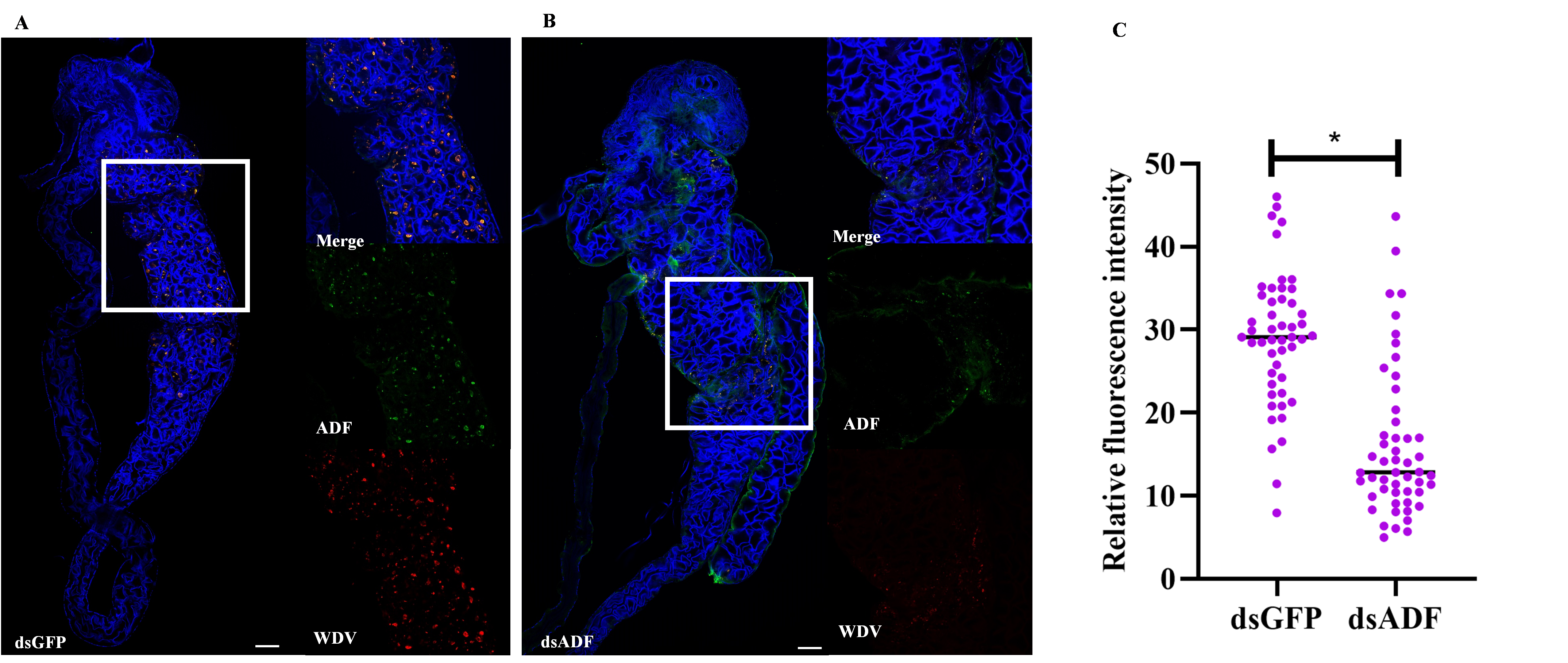


**Figure S6.** Confocal micrographs showing different WDV accumulation level in gut excised from leafhoppers after injection with ds*ADF* or ds*GFP*. Excised guts after injection with ds*GFP* (A) or ds*AD*F (B) were incubated with anti-ADF labeled with Dylight 488 (green) and anti-WDV labeled with CY3 (red). Size bar, 50 μm. Fluorescence of virus was analyzed using image J (C) .
