# Supplementary figures and images for: F‐actin dynamics in midgut cells enables virus persistence in vector insects
Source: Mol Plant Pathol. 2022 Sep 8;23(11):1671–85. doi: 10.1111/mpp.13260 (PMC9562576; doi:10.1111/mpp.13260)

**
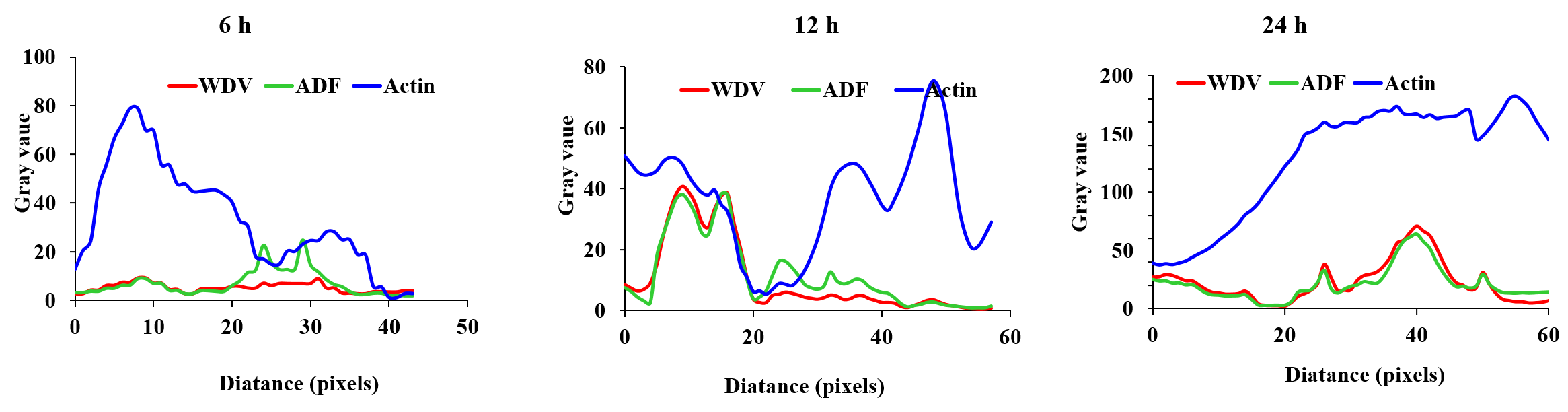
**

**Figure S7.** The plot profile presented was used to verify the visual colocalization in Figure 5G.

Supplement: Supplementary file 7 — Figure S7 The plot profile presented was used to verify the visual colocalization in Figure 5g [file MPP-23-1671-s005.docx]
