# Supplementary material for: F‐actin dynamics in midgut cells enables virus persistence in vector insects
Source: Mol Plant Pathol. 2022 Sep 8;23(11):1671–85. doi: 10.1111/mpp.13260 (PMC9562576; doi:10.1111/mpp.13260)
Supplement: Supplementary file 8 — Figure S8 WDV cannot change the F‐actin structure in gut cells of Schizaphis graminum. (a) Detection of WDV in S. graminum after feeding on WDV‐infected wheat seedlings for different AAPs using PCR with WDV‐specific primers. 1–4: 24‐h AAP, 5–8: 48‐h AAP, 9–12: 72‐h AAP, 13–16: 168‐h AAP. P: positive control, N: negative control. (b) LSCM images of the F‐actin structure in the excised gut from aphids after feeding on healthy wheat plants (left) and WDV‐infected wheat plants for 24 h (right) [file MPP-23-1671-s002.docx]

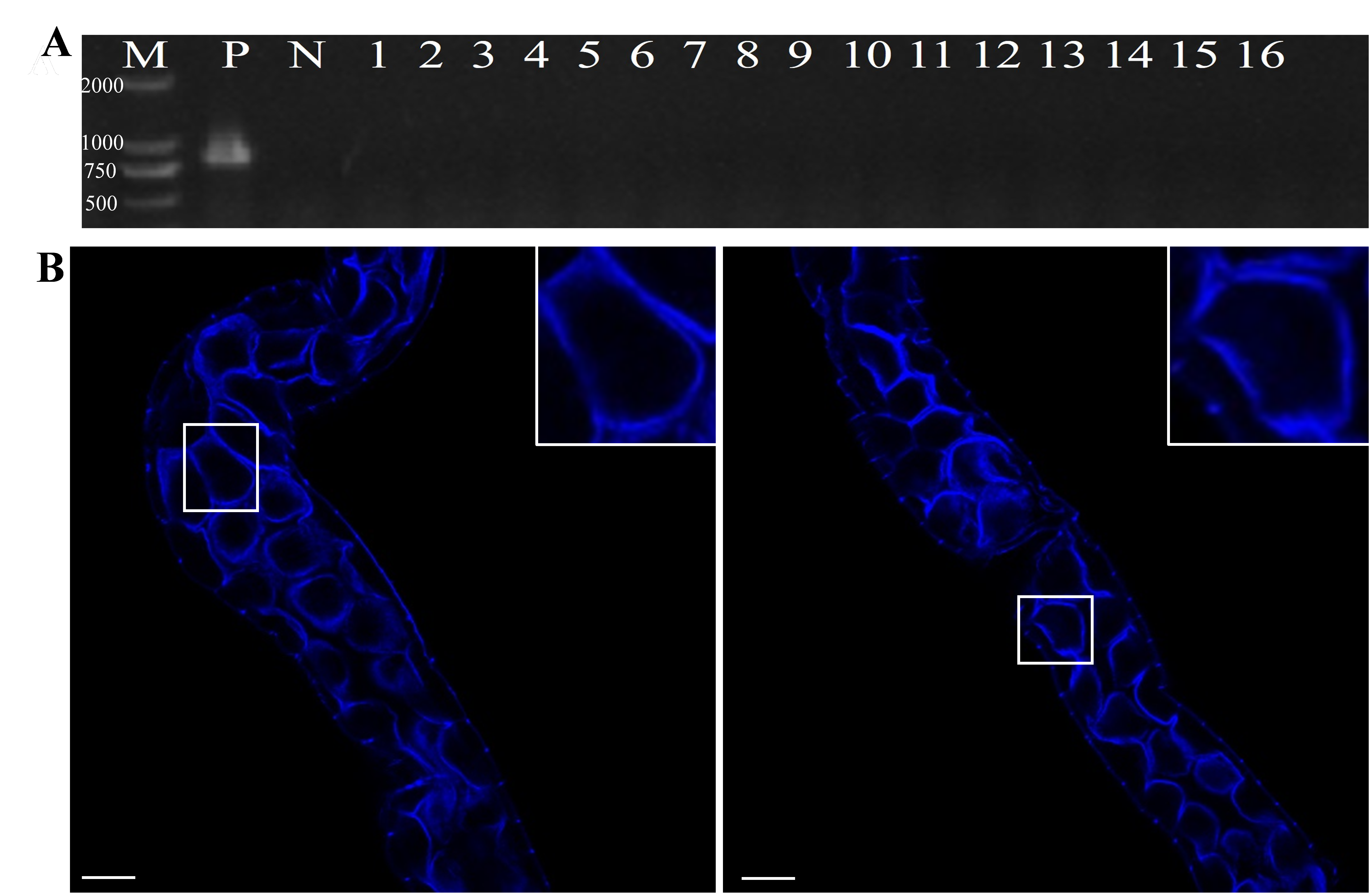


**Figure S8.** WDV cannot change structure of F-actin in gut cells of *S. graminum*.

(A) Detection of WDV in *S. graminum* after feeding on WDV-infected wheat seedling for different AAPs using PCR with the WDV-specific primer pairs. 1-4: 24 h AAP, 5-8: 48 h AAP, 9-12: 72 h AAP, 13-16: 168 h AAP. P: Posivive control. N: Negative control. (B) LSCM images of F-actin structure in excised gut from aphids after feeding on healthy wheat plants (left) and WDV-infected wheat plants for 24 h (right).
