# Supplementary material for: F‐actin dynamics in midgut cells enables virus persistence in vector insects
Source: Mol Plant Pathol. 2022 Sep 8;23(11):1671–85. doi: 10.1111/mpp.13260 (PMC9562576; doi:10.1111/mpp.13260)
Supplement: Supplementary file 9 — Table S1 Primers used in this study [file MPP-23-1671-s010.docx]

**Table S1.** Primers used in this study

| **Purpose** | Sequence (5’-3’) |
| --- | --- |
| **Gene clone** |  |
| ADF F | CTGACTGTTATAAGGTGATACGG |
| ADF R | CTGACTGTTATAAGGTGATACGG |
| **Yeast two-hybrid** |  |
| pPR3N ADF F | ATTAACAAGGCCATTACGGCCATGGCTTCCGGTGTGACGG |
| pPR3N ADF R | AACTGATTGGCCGAGGCGGCCCCTACTGCCGGTCAGTGGCAC |
| pDHB CP F | GGCCATTACGGCCATGGTGACCAACAAGGAC |
| pDHB CP R | GGCCGAGGCGGCCCCCACGCGTGCGTATAGGC |
| **RNAi** |  |
| dsADF F | ATTCTCTAGAAGCTTAATACGACTCACTATAGGGATGGCTTCCGGTGTGACGG |
| dsADF R | ATTCTCTAGAAGCTTAATACGACTCACTATAGGGCTACTGCCGGTCAGTGGCAC |
| dsGFP F | ATTCTCTAGAAGCTTAATACGACTCACTATAGGGGAGCTGTTCACCGGCATCGT |
| dsGFP R | ATTCCTCTAGAAGCTTAATACGACTCACTATAGGGCGATGGGGGTATTCTGCTGG |
| **WDV detection by PCR** | |
| CP F | ATGGTGACCAACAAGGAC |
| CP R | TAACACGCGTGCGTATAGGC |
| **Pull-down and Sf9 cell transfection** | |
| BamH I CP F | GGATCCATGGTGACCAACAAGGAC |
| Sal I CP R | GTCGACTAACACGCGTGCGTATAGGC |
| BamH I ADF F | GGATCCATGGCTTCCGGTGTGACGG |
| Sal I ADF R | GTCGACCTACTGCCGGTCAGTGGCAC |
| **qPCR** |  |
| ADF F | GAATATATAGGTGACCGCAAC |
| ADF R | CGATCGAAGGAGGAGGAGTA |
| REPS23 F | GGTGTTGAAGCTAAGCAGCC |
| REPS23 R | AGTTCAAACAACCGTCCCTG |
| CP F | CAACAAGGACTCCCGAGGTA |
| CP R | CAACAACATTGGTCCTGTCG |
| **RT-qPCR for BYDV-GAV** | |
| CP F | CAGGCAGGACTGAGGTATTCGTA |
| CP R | GGTTGCTGATTTTGAGATGGTGA |
