# Supplementary material for: F‐actin dynamics in midgut cells enables virus persistence in vector insects
Source: Mol Plant Pathol. 2022 Sep 8;23(11):1671–85. doi: 10.1111/mpp.13260 (PMC9562576; doi:10.1111/mpp.13260)
Supplement: Supplementary file 10 — Table S2 Transmission efficiency (%) of WDV by Psammotettix alienus after different AAPs [file MPP-23-1671-s003.docx]

**Table S2.** Transmission efficiency (%) of WDV by *P. alienus* after different acquisition access periods

|  | 4 d | 8 d | 12 d | 16 d | 20 d | 24 d | 28 d | 32 d | 36 d | 40 d |
| --- | --- | --- | --- | --- | --- | --- | --- | --- | --- | --- |
| 6 h | 66.67 a | 70 a | 70 a | 63.33 a | 56.67 a | 53.33 a | 43.33 a | 20 a | 10 a | 0 |
| 12 h | 80 ab | 83.33 ab | 73.33 ab | 70 ab | 56.67 a | 53.33 a | 56.67 b | 50 b | 20 a | 6.67 a |
| 24 h | 90 bc | 93.33 b | 83.33 ab | 80 b | 73.33 b | 73.33 b | 60 b | 53.33 b | 30 a | 10 a |
| 48 h | 96.67 c | 93.33 b | 86.67 b | 83.33 b | 76.67 b | 73.33 b | 63.33 b | 56.67 b | 23.33a | 13.33 a |

All the statistical analyses were performed with SPSS 20.0. Different letters in each of the numbers indicate significant differences. 6 h, 12 h, 24 h and 48h means different AAP. d: day.
